# Improved in vivo PET imaging of the adenosine A_2A_ receptor in the brain using [^18^F]FLUDA, a deuterated radiotracer with high metabolic stability

**DOI:** 10.1007/s00259-020-05164-4

**Published:** 2021-02-02

**Authors:** Thu Hang Lai, Magali Toussaint, Rodrigo Teodoro, Sladjana Dukić-Stefanović, Daniel Gündel, Friedrich-Alexander Ludwig, Barbara Wenzel, Susann Schröder, Bernhard Sattler, Rareş-Petru Moldovan, Björn H. Falkenburger, Osama Sabri, Winnie Deuther-Conrad, Peter Brust

**Affiliations:** 1 Department of Neuroradiopharmaceuticals, Institute of Radiopharmaceutical Cancer Research, Helmholtz-Zentrum Dresden-Rossendorf, Leipzig, Germany; 2 Department of Research and Development, ROTOP Pharmaka Ltd., Dresden, Germany; 3 Department of Nuclear Medicine, University Hospital Leipzig, Leipzig, Germany; 4 Department of Neurology, Dresden University Medical Center, Dresden, Germany

**Keywords:** Adenosine receptors, A_2A_ receptor, Neurodegeneration, Positron-emission tomography, Fluorine-18, FESCH

## Abstract

**Purpose:**

The adenosine A_2A_ receptor has emerged as a therapeutic target for multiple diseases, and thus the non-invasive imaging of the expression or occupancy of the A_2A_ receptor has potential to contribute to diagnosis and drug development. We aimed at the development of a metabolically stable A_2A_ receptor radiotracer and report herein the preclinical evaluation of [^18^F]**FLUDA**, a deuterated isotopologue of [^18^F]**FESCH**.

**Methods:**

[^18^F]**FLUDA** was synthesized by a two-step one-pot approach and evaluated in vitro by autoradiographic studies as well as in vivo by metabolism and dynamic PET/MRI studies in mice and piglets under baseline and blocking conditions. A single-dose toxicity study was performed in rats.

**Results:**

[^18^F]**FLUDA** was obtained with a radiochemical yield of 19% and molar activities of 72–180 GBq/μmol. Autoradiography proved A_2A_ receptor–specific accumulation of [^18^F]**FLUDA** in the striatum of a mouse and pig brain. In vivo evaluation in mice revealed improved stability of [^18^F]**FLUDA** compared to that of [^18^F]**FESCH**, resulting in the absence of brain-penetrant radiometabolites. Furthermore, the radiometabolites detected in piglets are expected to have a low tendency for brain penetration. PET/MRI studies confirmed high specific binding of [^18^F]**FLUDA** towards striatal A_2A_ receptor with a maximum specific-to-non-specific binding ratio in mice of 8.3. The toxicity study revealed no adverse effects of **FLUDA** up to 30 μg/kg, ~ 4000-fold the dose applied in human PET studies using [^18^F]**FLUDA**.

**Conclusions:**

The new radiotracer [^18^F]**FLUDA** is suitable to detect the availability of the A_2A_ receptor in the brain with high target specificity. It is regarded ready for human application.

**Supplementary Information:**

The online version contains supplementary material available at 10.1007/s00259-020-05164-4.

## Introduction

The signaling molecule adenosine is an important modulator of neurotransmission, regulating physiological processes such as sleep, motor activity, or sensorimotor gating [1]. Consequently, modulating adenosine signaling is an emerging treatment option for neuropsychiatric and neurodegenerative disorders [1]. Adenosine regulates neurotransmission of glutamate, acetylcholine, γ-aminobutyric acid, and dopamine using four G-protein-coupled plasma membrane receptors - adenosine A_1_, A_2A_, A_2B_, and A_3_ receptors [1]. Whereas A_1_, A_2B_ and A_3_ receptors are widely distributed throughout the brain, the A_2A_ receptor is specifically expressed at high densities in the dorsal and ventral striatum, the main input structures of basal ganglia circuitry [2]. The binding of adenosine to the A_2A_ receptor activates protein kinase A (PKA) via G protein–mediated stimulation of adenylyl cyclase and the corresponding increase in cyclic adenosine monophosphate (cAMP). In addition, PKA-independent pathways have also been reported [3, 4]. The A_2A_ receptor forms dimers with itself and with further G-protein-coupled receptors, in particular D_2_, mGluR_5_, CB_1_, and A_1_ [3], which causes changes in A_2A_ density in a wide range of neuropsychiatric and neurodegenerative diseases.

For instance, the measurement of mRNA levels indicated a reduced A_2A_ receptor density in the caudate and putamen of patients with Parkinson’s disease (PD) [5], while increased A_2A_ protein levels were found in PD patients with dyskinesias [6, 7] and in patients with Huntington’s disease (HD) [8]. Given that PD is characterized by a reduced mobility whereas PD patients with dyskinesias and HD patients show excessive movements, these findings collectively indicate that A_2A_ is an important reporter - and modulator - of mobility in human basal ganglia. Accordingly, the A_2A_ antagonist istradefylline has been approved for treatment of PD in the USA and in Japan [3, 9].

**Fig. 1:**
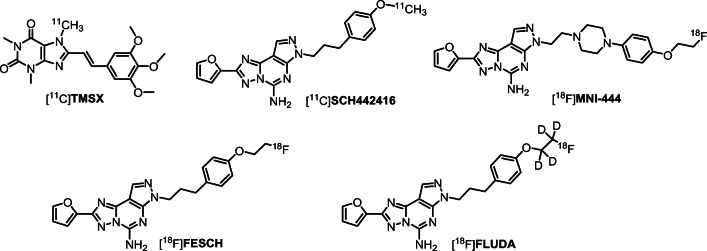
Representative radiotracers for PET imaging of the A_2A_ receptor and the herein reported [^18^F]**FLUDA**

Molecular imaging of the A_2A_ receptor by means of positron-emission tomography (PET) has the potential to quantitatively assess the receptor availability and changes thereof during the course of neuropsychiatric diseases’ pathological processes and determine optimal dosing regiments for drugs targeting A_2A_. Accordingly, research teams in both academia and industry have been working on the development of suitable PET radiotracers since more than 15 years. Initially, ^11^C-labeleld caffeine derivatives such as [^11^C]**KF17837** [10], [^11^C]**CSC** [11], or [^11^C]**KF21213** [12] were developed and investigated in animal models. With [^11^C]**SCH442416**, one of the first non-xanthine A_2A_ receptor antagonists has been proven as suitable for in vivo imaging [13]. The first studies in humans assessing the distribution of A_2A_ receptors in normal human brain with [^11^C]**KF18446** (also known as [^11^C]**TMSX**, Fig. 1) [14] as well as the occupancy of the A_2A_ receptor of the targeted drug candidate vipadenant with [^11^C]**SCH442416** (Fig. 1) were published about 10 years ago [15]. At about the same time, the first ^18^F-labeled radiotracers were reported, e.g., analogs of **SCH442416**, such as [^18^F]**MRS5425**, (also known as [^18^F]**FESCH**, Fig. 1) [16, 17]. [^18^F]**MNI-444** (Fig. 1) is another PET radiotracer that was used for imaging of the A_2A_ receptor in healthy human subjects [18]. To assess the suitability of A_2A_ receptor PET for the assessment of changes in the availability of A_2A_/D_2_ heterodimeric receptors in neurodegenerative diseases, our group performed dynamic PET studies in a rotenone-based mouse model of Parkinson’s disease with [^18^F]**FESCH**. However, the study yielded inconclusive data, at least in part due to the penetration of a non-negligible fraction of a single radiometabolite into the mouse’ brain [19]. Therefore, we intended to enhance the metabolic stability of [^18^F]**FESCH** by developing a deuterated isotopologue, **FLUDA**. To test the hypothesis that a selective substitution with deuterium improves the imaging properties of the novel A_2A_ receptor–specific radiotracer [^18^F]**FLUDA** (Fig. 1), we performed a series of preclinical animal studies including in vivo metabolism and dynamic PET/MRI investigations in mouse and dynamic PET investigations in piglets to investigate target specificity and pharmacokinetics in different species. To further support the transfer of [^18^F]**FLUDA** to first-in-human studies, a preclinical acute toxicity study in rats has been commissioned as well.

## Materials and methods

The full description of all procedures is provided in the Supplementary information (SI).

### Chemical synthesis

**Scheme 1:**
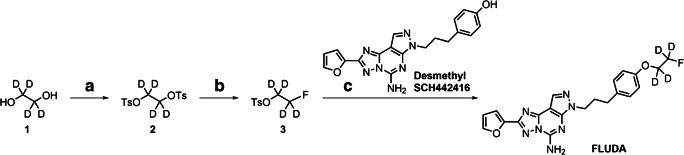
Synthesis of **FLUDA**, reagents and conditions. **a** *p*-TsCl, NEt_3_, CH_2_Cl_2_, 0 °C, 3 h, 68% yield. **b** TBAF, MeCN/THF, 90 °C, 15 min, 36% yield. **c Desmethyl SCH442416**, Cs_2_CO_3_, MeOH, microwave heating (1 h, 100 °C, 100 W), 37% yield

**FLUDA** was synthesized by a microwave-assisted alkylation reaction of 4-[3-(5-amino-2-furan-2-yl-pyrazolo[4,3*-e*][1,2,4]triazolo[1,5-*c*]pyrimidin-7-yl)-propyl]-phenol (**desmethyl SCH442416**) and 2-fluoroethyl-1,1,2,2-*d*_4_ 4-methylbenzenesulfonate (**3**), that was previously prepared by tosylation of the commercially available ethane-*d*_4_-1,2-diol (**1**) and fluorination of ethane-1,2-diyl-*d*_4_ bis(4-methylbenzenesulfonate) (**2**, Scheme 1).

### Radiosynthesis

**Fig. 2:**
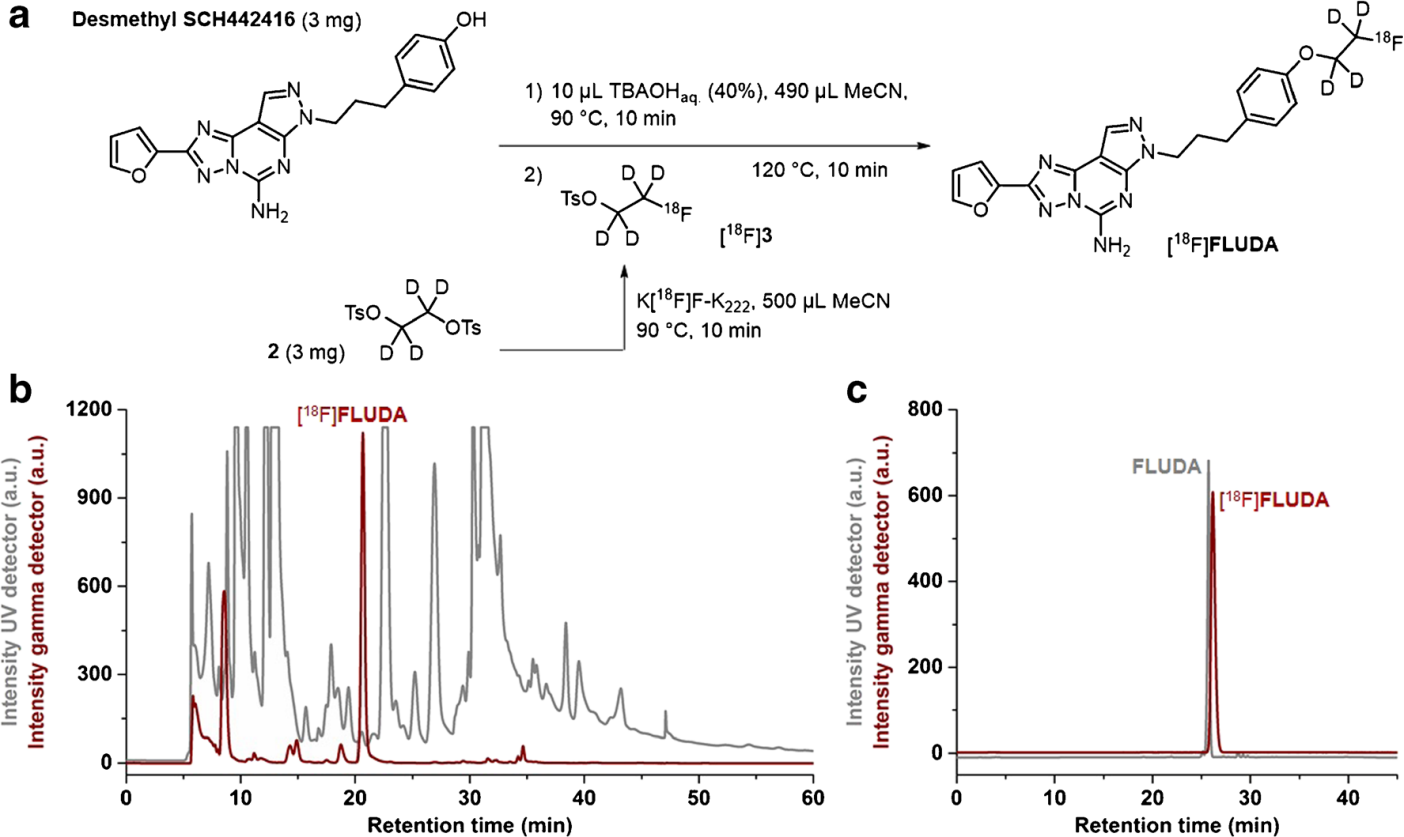
**a** Scheme of the two-step one-pot procedure for the radiosynthesis of [^18^F]**FLUDA. b** Representative chromatograms of isolation of [^18^F]**FLUDA** by semi-preparative HPLC. **c** Identification of [^18^F]**FLUDA**. HPLC chromatograms obtained by co-injection with the reference compound **FLUDA**

[^18^F]**FLUDA** was prepared by a two-step one-pot manual radiosynthesis of the tosylate precursor **2** and the phenol precursor **desmethyl SCH442416** with [^18^F]fluoride in anhydrous acetonitrile (MeCN) in the presence of potassium carbonate and Kryptofix 222 (K_222_) according to our optimized procedure for the radiosynthesis of [^18^F]**FESCH** (Fig. 2a) [19].

### Physiochemical properties

The chemical stability of [^18^F]**FLUDA** was proven in saline, phosphate-buffered saline (PBS, pH 7.4) and *n*-octanol by incubation at 37 °C up to 60 min followed by radio-TLC and radio-HPLC analyses. The log*D*_7.4_ value was experimentally determined by the conventional shake-flask method using *n*-octanol and PBS as partition system (*n* = 4).

### Animals

The animal experiments were performed with female CD-1 mice (10–12 weeks, 26–38 g) obtained from the Medizinisch-Experimentelles Zentrum (MEZ) at Universität Leipzig (Leipzig; Germany) and female piglets (6–12 weeks old, 14–18 kg) obtained from the Lehr-und Versuchsgut of the Faculty of Veterinary Medicine at Universität Leipzig (LVG; Oberholz; Germany).

### In vitro binding assays

The test compounds (10 mM stock in DMSO) were incubated with crude cell membrane homogenates obtained from CHO cells stably transfected with human A_2A_ receptor or human A_1_ receptor and with A_2A_ receptor–specific [^3^H]**ZM241385** or A_1_ receptor–specific [^3^H]**DPCPX** in an incubation buffer at room temperature (RT). The IC_50_ values were determined by non-linear regression analysis with GraphPad Prism 4.1 (GraphPad Inc.; La Jolla; CA), and *K*_i_ values estimated according to the Cheng-Prusoff equation with *K*_D,ZM241385_ = 0.8 nM and *K*_D,DPCPX_ = 0.45 nM.

### In vitro autoradiography

Cryosections from mouse and piglet brain were thawed, dried, and pre-incubated with buffer containing adenosine deaminase (ADA), and then with buffer containing ADA and [^18^F]**FLUDA** alone or together with 10 μM **ZM241385** to assess non-specific binding of [^18^F]**FLUDA** or together with different concentrations of test compounds to estimate their binding affinity towards the A_2A_ receptor. After washing and drying, the slides were exposed to a phosphor imager plate, and after scanning, the autoradiographic images were analyzed with the AIDA 2.31 software and inhibition curves created with GraphPad Prism 4.1 (GraphPad Inc., La Jolla, CA).

### In vivo metabolism studies

In mice, [^18^F]**FLUDA** was administered i.v. as a bolus in the tail vein of awake animals (~ 36 MBq, *n* = 3). After 15 min, the animals were slightly anesthetized by isoflurane inhalation, and blood samples were taken by retro-orbital bleeding. The blood plasma was obtained by centrifugation. The brain was isolated immediately after cervical dislocation and homogenized in water. For analysis by RP-HPLC, brain homogenates and blood plasma were mixed with the 4-fold volumes of acetone/water (4/1; *v/v*), precipitated proteins removed by centrifugation, the supernatants were concentrated and analyzed by analytical RP-HPLC. For analysis by micellar-HPLC (MLC), brain homogenates and blood plasma were mixed with equal volumes of aqueous sodium dodecyl sulfate and directly injected into the MLC system.

In piglets, [^18^F]**FLUDA** was administered i.v. as a bolus in the auricular vein of the anesthetized animals (~ 203 MBq, *n* = 2). Arterial blood was sampled over 120 min and the blood plasma was obtained by centrifugation. For analysis by semi-preparative RP-HPLC, blood plasma was mixed with the twofold volumes of acetone/water (4/1; *v/v*), precipitated proteins were removed by centrifugation, and the supernatants were concentrated and analyzed by semi-preparative RP-HPLC.

### Dynamic PET studies in mice

PET/MRI scans were performed using a preclinical PET/MRI system (PET/MRI 1Tesla; nanoScan^®^; MEDISO Medical Imaging Systems; Budapest; Hungary) in CD-1 mice under baseline (*n* = 4), control (vehicle, *n* = 8), and blocking (pre-administration of 2.5 mg/kg tozadenant (also known as **SYN-115**) or 1.0 mg/kg istradefylline (also known as **KW-6002**) i.v. 15 to 8 min before radiotracer *n* = 4, respectively) conditions. Dynamic whole-body animal PET scans were acquired during 60 min after i.v. administration of [^18^F]**FLUDA** (3.1–9.7 MBq, 0.7–4.5 nmol/kg). T1-weighted imaging was performed afterwards for anatomical orientation and attenuation correction. After reconstruction, PET images were analyzed in PMOD 3.9 (PMOD technologies LLC; Zurich; Switzerland), and volumes of interest were applied to the PET series to extract time-activity curves (TACs). TACs were expressed in standardized uptake value (SUV).

### PET studies in piglets

PET scans were obtained on a clinical PET-System (ECAT Exact HR+; Siemens Healthcare GmbH; Erlangen; Germany) in piglets under control (vehicle, *n* = 1) and blocking conditions (2.5 mg tozadenant /kg i.v. 15 min before radiotracer followed by continuous infusion at 0.9 mg/kg/h for the duration of the study, *n* = 1). Dynamic PET scans were acquired during 90 min after i.v. administration of [^18^F]**FLUDA** (178–229 MBq; 0.08–0.16 fmol/kg, for control and blocking conditions respectively). After reconstruction, PET images were analyzed in PMOD 3.9 (PMOD technologies LLC; Zurich; Switzerland).

### Toxicity studies in rats

The extended single-dose toxicity studies of **FLUDA** in male (*n* = 45) and female (*n* = 45) outbreed Wistar rats were performed in the Biological Testing Laboratory (BTL) in Russia (Study Number 678/19). The test item **FLUDA** was administered by a single bolus i.v. injection at doses of 6 and 30 μg/kg body weight (bw). Mortality, clinical pathology parameters (hematology and serum chemistry), organ weights, and microscopic tissue parameters were investigated 24 h and 2 weeks after treatment.

## Results

### Synthesis of the reference compound and affinity investigation

The deuterated derivative of **FESCH** was obtained by the use of a deuterated fluoroethyl building block according to the synthesis described in Scheme 1. Binding studies were performed by competitive radiotracer binding assays and revealed high-affinity binding of **FLUDA** towards the human A_2A_ receptor with a *K*_i_ value of 0.74 ± 0.26 nM and negligible binding towards the human A_1_ receptor (*K*_i_ > 1 μM).

### Radiosynthesis, lipophilicity, and chemical stability

[^18^F]**FLUDA** was obtained by a one-pot two-step radiofluorination as shown in Fig. 2 with a radiochemical yield of 19 ± 3% (end of bombardment = EOB), a radiochemical purity of ≥ 99%, and molar activities in the range of 72–180 GBq/μmol (end of synthesis = EOS) within a total synthesis time of 102 ± 4 min (*n* = 9). The shake-flask method was used to determine the log*D*_7.4_ value of [^18^F]**FLUDA** (2.01 ± 0.07). The incubation of [^18^F]**FLUDA** in saline, PBS (pH 7.4), and *n*-octanol has shown no degradation or defluorination.

### In vivo metabolism

**Fig. 3:**
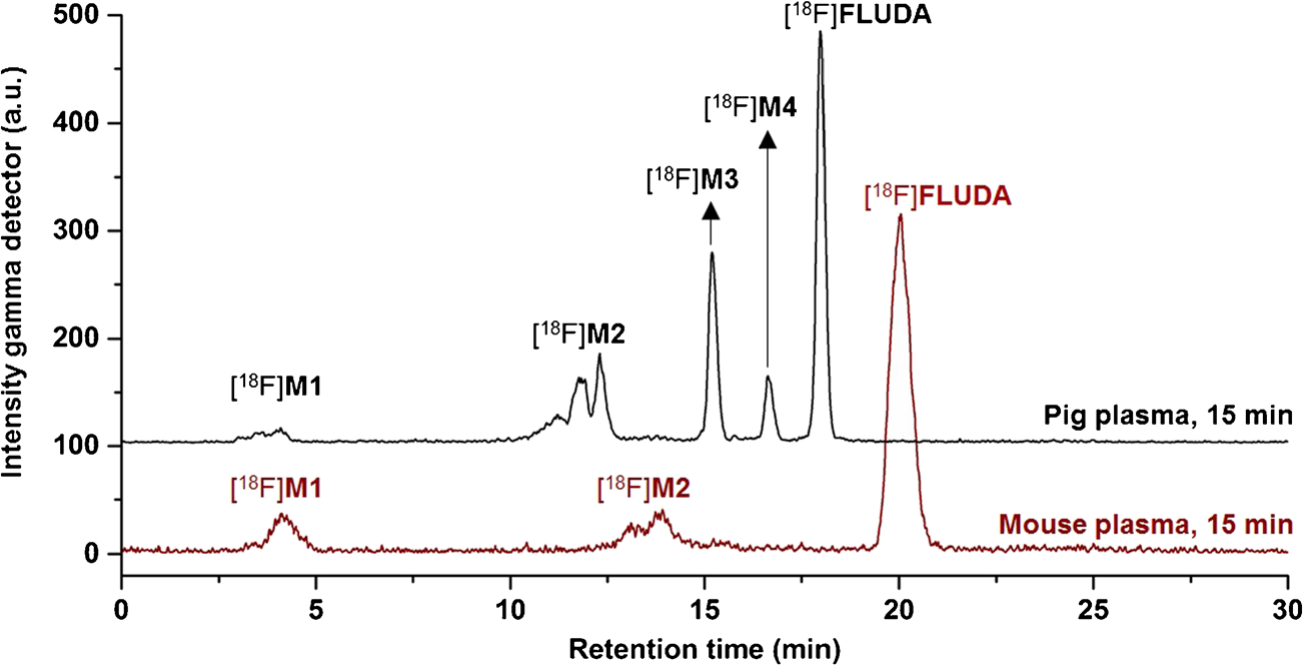
Representative RP-HPLC radiochromatograms of plasma samples after administration of [^18^F]**FLUDA** to a mouse and piglet

At 15 min after i.v. injection of [^18^F]**FLUDA** in mice and analysis, the parent fraction accounted for about 100% (RP-HPLC, recovery: 98%) and 95% (MLC) in brain (Fig. S2); and 71% (RP-HPLC, recovery: 86%, Fig. 3) and 56% (MLC, Fig. S1) in plasma. In piglets, the parent fraction accounted for about 47% in plasma at 16 min p.i (recovery: 89%, Fig. S1). The metabolic pattern in mice contained two fractions of radiometabolites, [^18^F]**M1** and [^18^F]**M2**, not able to cross the blood-brain barrier (BBB). In contrast to the in vivo stability of [^18^F]**FLUDA** in mice, the plasma samples of piglets contained two additional radiometabolites, [^18^F]**M3** and [^18^F]**M4**, supposed to have a similar structure to [^18^F]**FLUDA** based on their chromatographic behavior as shown in Fig. 3. Due to the PET studies in piglets (Fig. 7), these two radiometabolites are not expected to cross the BBB.

### Quantitative in vitro autoradiography

**Fig. 4:**
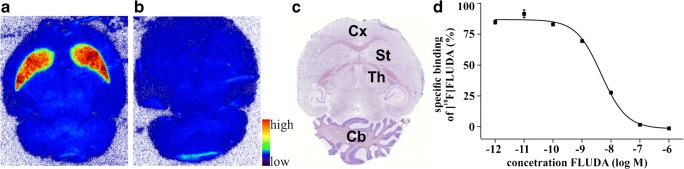
Representative in vitro autoradiographic images of the binding pattern of [^18^F]**FLUDA** (0.93 nM) in horizontal mouse brain slices. The highest accumulation of activity in the striatum (**a**, red). The binding is completely blocked by co-administration of 10 μM of the A_2A_ receptor antagonist **ZM241385** (**b**). For annotation of the brain regions, the slices were Nissl-stained after autoradiography (**c**). St striatum, Cb cerebellum, and Cx cortex, Th thalamus. Representative competition curve (**d**). *K*_D_ and *B*_max_ calculated from the homologous completion of [^18^F]**FLUDA** with **FLUDA** (Cheng-Prusoff equation *K*_D_ = IC_50_− [[^18^F]**FLUDA**] and *B*_max_ = top – bottom ∙ (*K*_D_ + [[^18^F]**FLUDA**])/[[^18^F]**FLUDA**] [20].

**Fig. 5:**
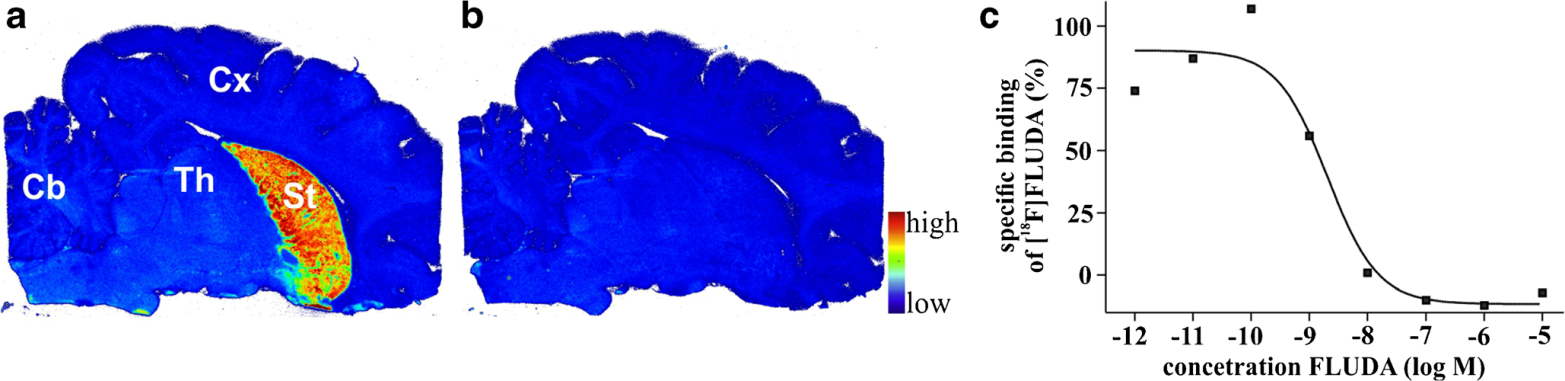
In vitro autoradiographic images of the binding pattern of [^18^F]**FLUDA** (0.64 nM) in sagittal pig brain slices. The highest accumulation of activity is in the striatum (**a**, red, St striatum, Cb cerebellum, Cx cortex, and Th thalamus). The binding is completely blocked by co-administration of 10 μM of the A_2A_ receptor antagonist **ZM241385** (**b**). Homologous competition curve (**c**)

The binding pattern of [^18^F]**FLUDA** in mouse brain is heterogeneous with the highest density of binding sites in the striatum, an A_2A_ receptor–rich region. In A_2A_ receptor–poor regions, such as cerebellum, midbrain, cortex, or thalamus, only negligible binding was detected (Fig. 4a, Fig. S3). About 90% of the binding of ~ 1 nM [^18^F]**FLUDA** in mouse striatum could be displaced by co-incubation with 10 μM **ZM241385** (Fig. 4b). The binding sites in striatum were further characterized by saturation experiments which revealed a *K*_D_ value of 4.30 ± 0.73 nM and a *B*_max_ value of 556 ± 143 fmol/mg wet weight (Fig. 4d). Comparable results were obtained in a single experiment performed in cryosections of the pig brain to promote the preclinical evaluation of [^18^F]**FLUDA** in larger species (Fig. 5). The nearly exclusive binding of [^18^F]**FLUDA** in the striatum (Fig. 5a) was completely blocked by co-administration of **ZM241385** (Fig. 5b). By homologous competition, the binding of **FLUDA** in the pig striatum was characterized with a *K*_D_ value of 0.68 nM and a *B*_max_ value of 218 fmol/mg wet weight (Fig. 5c).

### Dynamic PET studies in mice

**Fig. 6:**
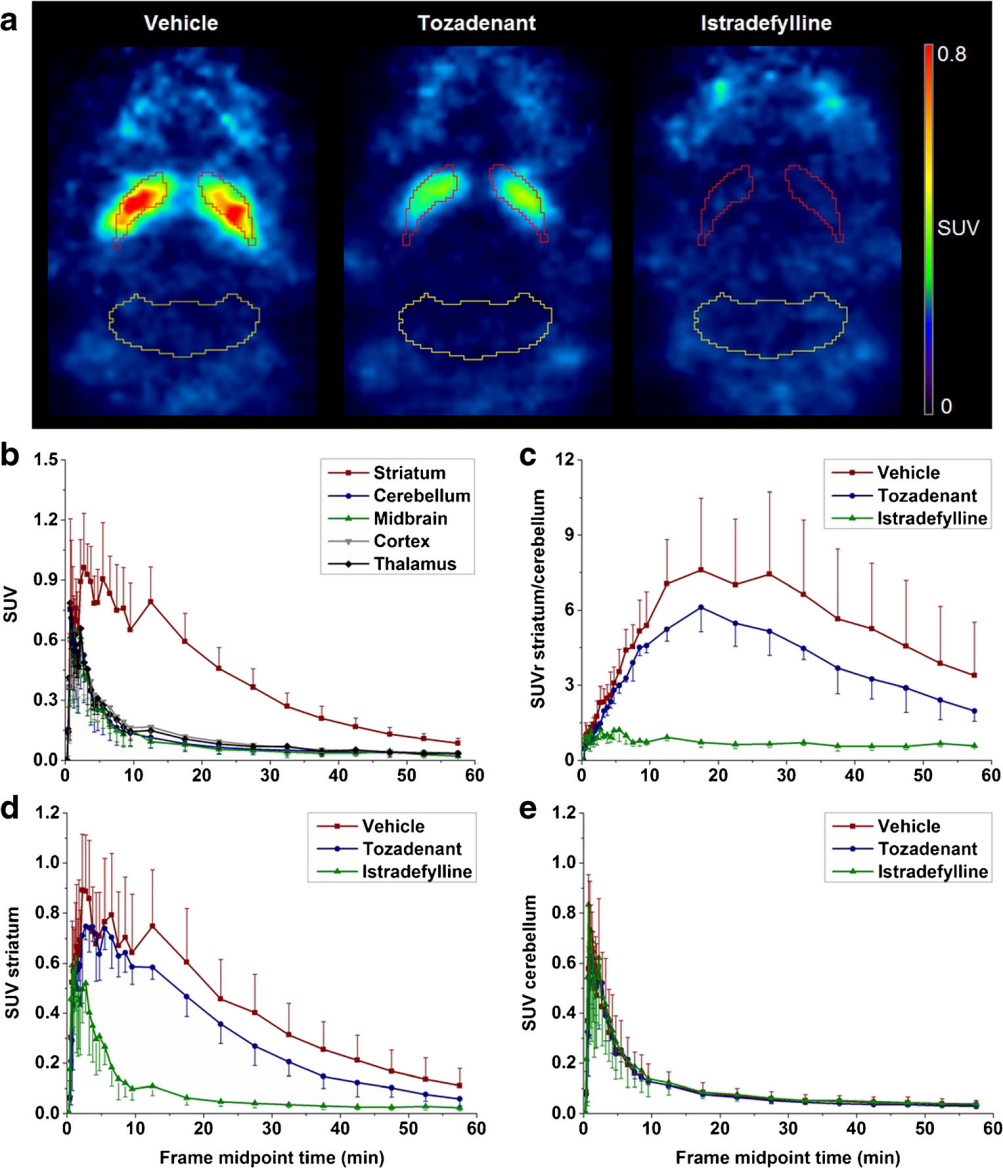
**a** Representative horizontal PET images (0–60 min) of [^18^F]**FLUDA** in the brain of CD-1 mice (striatum: red, cerebellum: yellow). **b** TACs at baseline for CD-1 mice (*n* = 4) in different brain regions after injection of [^18^F]**FLUDA**; TACs of **c** SUVr_St/Cb_, **d** SUV striatum, and **e** SUV cerebellum: after pre-treatment with vehicle (red square, *n* = 8), tozadenant (2.5 mg/kg bw, blue circle, *n* = 4) and istradefylline (1.0 mg/kg bw, green triangle, *n* = 4)

Representative PET images of [^18^F]**FLUDA** in a mouse brain are shown in Fig. 6a. Corresponding to the in vitro autoradiography, the highest uptake was detected in the A_2A_ receptor–rich striatum. Also, the negligible accumulation of activity in A_2A_ receptor–poor regions, such as cerebellum, is in agreement with the in vitro data. These general findings are confirmed by the analysis of the PET-derived regional time-activity curves (TACs) presented in Fig. 6b. In fact, a high specific-to-non-specific binding ratio was reached, with a maximum SUVr_St/Cb_ of 8.3 reflecting a high target selectivity (Fig. 6c). Furthermore, the continuous washout of activity from all brain regions confirms the absence of brain-penetrant radiometabolites. To validate the target specificity of [^18^F]**FLUDA** in vivo, the A_2A_ receptor antagonist istradefylline (1 mg/kg) or tozadenant (2.5 mg/kg) were administered i.v. at 10 min before radiotracer injection. Maximum blocking was obtained by pre-treatment of istradefylline as reflected by the striatal SUV being similar to the cerebellar SUV under blocking conditions during the entire period of investigation (Fig. 6d–e). By tozadenant pre-treatment, the accumulation of activity in the striatum was reduced by 23.5% in comparison to the control (according to the area under the curve (AUC); AUC_vehicle_: 24.9 ± 8.7 SUV ∙ min; AUC_tozadenant_: 19.0 ± 4.3 SUV ∙ min from 0 to 60 min; Fig. S8). The activity accumulation in the cerebellum was not significantly affected by the blocking compound (Fig. 6e; AUC_vehicle_: 5.9 ± 3.0 SUV ∙ min; AUC_tozadenant_: 5.4 ± 1.3 SUV ∙ min from 0 to 60 min; Fig. S8), confirming the target specificity of [^18^F]**FLUDA** and indicating the suitability of the cerebellum as a reference region for A_2A_ receptor imaging.

Further analysis of the whole-body PET data obtained in control (vehicle-treated) animals revealed a high initial uptake in the small intestine and the liver, followed by a pronounced accumulation of activity in the former, along with a stable uptake in the kidney over the whole scanning time, indicating that [^18^F]**FLUDA** and/or its metabolites mainly undergo hepatobiliary excretion (Table S2; Fig. S9).

### Dynamic PET studies in piglets

**Fig. 7:**
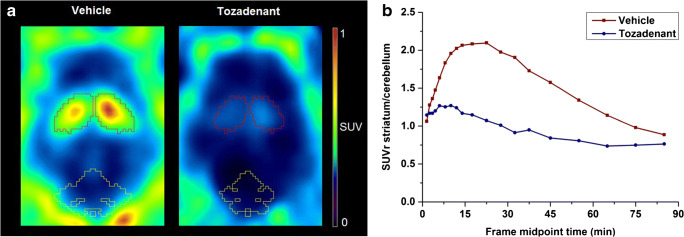
**a** Representative horizontal PET images (0–60 min) of [^18^F]**FLUDA** in the brain of piglets (striatum: red, cerebellum: yellow). **b** TACs of SUVr_St/Cb_ of [^18^F]**FLUDA** in the pig brain after administration of vehicle (red square, *n* = 1) or blocking with tozadenant (blue circle, *n* = 1)

To further assist the transfer of [^18^F]**FLUDA** to clinical studies, we performed a pilot study to evaluate the pharmacokinetic profile of [^18^F]**FLUDA** by dynamic PET in piglets. The activity distribution in the brain of a control subject resembles the findings obtained in mouse—strong and nearly exclusive accumulation in the striatum and low signal in all other brain regions (Fig. 7a). The pre-administration of tozadenant (2.5 mg/kg) at 15 min before radiotracer followed by continuous infusion inhibited the accumulation of activity in the striatum by 50% (AUC_vehicle_: 42.1 SUV ∙ min; AUC_tozadenant_: 21.0 SUV ∙ min from 0 to 60 min), and in A_2A_ receptor–poor regions, such as cerebellum by 17% (AUC_vehicle_: 24.0 SUV ∙ min; AUC_tozadenant_: 19.8 SUV ∙ min from 0 to 60 min; Fig. 7b).

### Single-dose toxicity study

In an extended single-dose toxicity study in rats, administration of **FLUDA** up to 30 μg/kg body weight, which is about 4000 times the estimated human dose and thus in accordance with the ICH guideline M3(R2), which recommends a minimum of a 50-fold the clinical exposure as the maximum dose for general toxicity studies in any species, did not cause any conspicuous features related to the hematology, clinical chemistry, necropsy, and histopathology analyses.

## Discussion

The selective deuteration of the known A_2A_ receptor radiotracer [^18^F]**FESCH** significantly improved the metabolic stability of the A_2A_ receptor PET ligand [^18^F]**FLUDA**. Furthermore, the preclinical evaluation of [^18^F]**FLUDA** in both small and larger animals demonstrates high specific binding towards the adenosine A_2A_ receptor in the brain, indicating the suitability of [^18^F]**FLUDA** for first-in-human studies.

In comparison to [^18^F]**FESCH**, [^18^F]**FLUDA** is much less susceptible to biotransformation. This is reflected by the much higher amount of the parent fraction in plasma at 15 min after i.v. injection in mice (41% [19] vs. 71%). The activity accumulating in the brain after i.v. injection of [^18^F]**FLUDA** corresponds almost completely to the parent compound. This finding is supported by the profile of the region-specific TACs obtained in dynamic PET studies with [^18^F]**FLUDA** in both mice (Fig. 6) and piglets (Fig. 7), which do not indicate any confounding accumulation of radiometabolites in the brain. In summary, deuteration of the fluoroethoxy group of [^18^F]**FLUDA** eliminates the main obstacle observed in the investigation of disease-related changes of the A_2A_ receptor availability in preclinical studies using [^18^F]**FESCH**: significant amounts of brain-penetrant radiometabolites.

With respect to further more general properties such as affinity, selectivity, blood-brain barrier permeability, and clearance, [^18^F]**FLUDA** retains the positive characteristics of [^18^F]**FESCH** [16, 17, 19, 21]. The affinity of [^18/19^F]**FLUDA** towards human and mouse A_2A_ receptors (*K*_i_ ~ 1 nM vs*. K*_D_ ~ 4 nM) corresponds well to the results obtained for [^18/19^F]**FESCH** (*K*_i_ ~ 1 nM vs. *K*_D_ ~ 5 nM; [19]) including a slight discrepancy between the two species that was reported previously (Table S1, Figs. S3, S4, S5, S6, S7) [22]. Regarding specificity, the accumulation of [^18^F]**FLUDA** resembles the expression pattern of the A_2A_ receptor protein in both mouse brain and pig brain and both in vitro and in vivo (Figs. 4, 5, 6, and 7). Moreover, the accumulation of [^18^F]**FLUDA** can be blocked completely by structurally different selective A_2A_ receptor antagonists (Figs. 6 and 7). The presence of an A_2A_ receptor antagonist inhibited the accumulation of activity in the striatum almost completely but did not affect any other brain region in mice and affected the cerebellum by 17% in piglet. However, the high specificity of [^18^F]**FLUDA**, as well as the absence of effect of blocking compound on cerebellum activity accumulation in mice studies, suggests that this effect on the cerebellum of piglet is most likely due to inter-individual variability, and would need a larger-scale study to investigate its significance. Collectively, these findings indicate highly specific in vivo binding of [^18^F]**FLUDA** in the striata in both species.

The differences in the efficacy of the two A_2A_ receptor ligands istradefylline and tozadenant to inhibit the binding of [^18^F]**FLUDA** in vitro and in vivo can be explained by known differences in the properties of these antagonists, in particular the remarkably low affinity of tozadenant towards mouse A_2A_ receptors, which we determined in-house by [^18^F]**FLUDA** autoradiography (istradefylline: *K*_i_ (mouse A_2A_ receptor) ~ 60 nM, *K*_i_ (piglet A_2A_ receptor) ~ 15 nM; tozadenant: *K*_i_ (mouse A_2A_ receptor) ~ 250 nM, *K*_i_ (piglet A_2A_ receptor) ~ 10 nM; Table S1). Most of the preclinical studies that evaluated the specificity of A_2A_ receptor–targeting PET radiotracers use istradefylline [17, 21, 23], whereas tozadenant has only been applied, to the best of our knowledge, in a single study in rhesus monkeys [24]. A head-to-head comparison as presented here for mice has not been reported so far. The higher efficacy of tozadenant in vivo in piglet in comparison to mouse (~ 50% displacement vs. ~ 25% displacement at 2.5 mg/kg followed by 0.9 mg/kg/h infusion and 2.5 mg/kg, respectively) can be explained by the dose-response results reported for the occupancy of [^18^F]**MNI-444**-labeled binding sites by tozadenant (47% vs. 95% at 1.5 mg/kg and 10.5 mg/kg, respectively; [24]). The observed difference in the maximum striatum-to-cerebellum ratio between mice (8.3) and piglet (2.1) is regarded to be caused by the different A_2A_ receptor densities in the striatum, which were autoradiographically determined to be twofold higher in mice than in pigs [25].

By taking into consideration the density of the A_2A_ receptor in the human striatum under physiologic conditions (e.g., 260–444 fmol/mg protein [26, 27]), we assume that the binding potential of [^18^F]**FLUDA** is suitable to quantify the receptor availability in humans. The highly specific binding of [^18^F]**FLUDA** towards the A_2A_ receptor was confirmed by blocking studies conducted in vitro (Figs. 4 and 5) and in vivo (Figs. 6 and 7). The presence of A_2A_ receptor antagonists inhibited the accumulation of activity in striatum almost completely but did not affect any other brain region. Accordingly, from the herein presented exploratory study, we assume that similar to the already clinically applied non-deuterated [^18^F]**FESCH** or [^11^C]**preladenant**, the quantification and kinetic modeling of PET data obtained with [^18^F]**FLUDA** might be facilitated by the use of the cerebellum as a reference region [21, 28]. However, full kinetic modeling with arterial input functions, ideally performed in a large animal and under conditions comparable to human studies, is required to validate the use of such region for the quantification of the availability of the A_2A_ receptor by [^18^F]**FLUDA** PET.

## Conclusion

[^18^F]**FLUDA** is a new A_2A_ receptor–targeting PET radiotracer with promising preclinical results for clinical translation. The radiotracer can be prepared with high molar activities and in reasonable radiochemical yield (manuscript reporting automated synthesis under preparation). Highly affine and specific binding of [^18^F]**FLUDA** in vivo was demonstrated by PET studies performed in different species. The selective deuteration resulted in a high metabolic stability, and the identification of cerebellum as a reference region is assumed to facilitate the quantification of the availability of the A_2A_ receptor in the brain and disease-related changes thereof in clinical routine. Furthermore, the results of a toxicity and a dosimetry study [29] indicate that the use of [^18^F]**FLUDA** in first-in-human imaging studies is safe.

## Supplementary Information

ESM 1 (PDF 1.95 mb)

## Data Availability

The datasets generated during and/or analyzed during the current study are available from the corresponding author on reasonable request.
