# Supplementary material for: Improved in vivo PET imaging of the adenosine A2A receptor in the brain using [18F]FLUDA, a deuterated radiotracer with high metabolic stability
Source: Eur J Nucl Med Mol Imaging. 2021 Feb 2;48(9):2727–36. doi: 10.1007/s00259-020-05164-4 (PMC8263428; doi:10.1007/s00259-020-05164-4)
Supplement: Supplementary file 1 — (PDF 1.95 mb) [file 259_2020_5164_MOESM1_ESM.pdf]

Supplementary Material

Improved *in vivo* PET imaging of the adenosine A_{2A} receptor in the brain using $[^{18}F]$FLUDA, a deuterated radiotracer with high metabolic stability

Thu Hang Lai[1,2‡*], Magali Toussaint[1,‡,*], Rodrigo Teodoro[1], Sladjana Dukić-Stefanović[1], Daniel Gündel[1], Friedrich-Alexander Ludwig[1], Barbara Wenzel[1], Susann Schröder[2], Bernhard Sattler[3], Rareş-Petru Moldovan[1], Björn H. Falkenburger[4], Osama Sabri[3], Winnie Deuther-Conrad[1,‡], Peter Brust[1,‡]

[1]Helmholtz-Zentrum Dresden-Rossendorf, Institute of Radiopharmaceutical Cancer Research, Department of Neuroradiopharmaceuticals, Leipzig, Germany; [2]ROTOP Pharmaka Ltd., Department of Research and Development, Dresden, Germany; [3]Department of Nuclear Medicine, University Hospital Leipzig, Leipzig, Germany; [4]Department of Neurology, Dresden University Medical Center, Dresden, Germany.

[‡]These authors contributed equally to this work

[*]**Corresponding authors:**

Thu Hang Lai, t.lai@hzdr.de

Magali Toussaint, m.toussaint@hzdr.de

Contents

Material and methods 4

General 4

Chemical synthesis 4

7-(3-(4-(2-Fluoroethoxy-1,1,2,2-*d*$_4$)phenyl)propyl)-2-(furan-2-yl)-7*H*-pyrazolo-[4,3-*e*][1,2,4]triazolo[1,5-*c*]pyrimidin-5-amine (**FLUDA**) 4

Radiosynthesis of [^{18}F]FLUDA 6

Quality control 7

Physiochemical properties 7

Biological evaluation 8

In vitro binding assays 8

In vitro autoradiography 8

In vivo metabolism in mice 9

In vivo metabolism in piglets 10

Dynamic PET studies in mice 11

PET studies in piglets 11

Toxicity studies in rats 12

Supporting tables and figures 13

Tab. S1: Affinities of A_{2A} receptor antagonists in radioligand binding studies and autoradiographic studies at A_{2A} receptor. 13

Fig. S1: Parent radiotracer fractions profile of plasma samples after administration of [^{18}F]**FLUDA** to a piglet. 14

Fig. S2: Representative (A) RP-HPLC and (B) MLC radio-chromatograms of a mouse brain and plasma sample 15 min p.i. of [^{18}F]FLUDA. 14

Fig. S3: Representative *in vitro* autoradiographic images of the binding pattern of [^{18}F]**FLUDA** in horizontal mouse brain slices. The highest accumulation of activity in the striatum (A, red; St: striatum, Cb: cerebellum, Cx: cortex, Th: thalamus). Self-blocking by co-administration of 10 nM ZM241385 (B). The binding is completely blocked by co-administration of 10 µM of the A_{2A} receptor antagonist istradefylline (C). Representative competition curve (D). 15

Fig. S4: Representative *in vitro* autoradiographic images of the binding pattern of [^{18}F]**FLUDA** in horizontal mouse brain slices. The highest accumulation of activity in the striatum (A, red; St: striatum, Cb: cerebellum, Cx: cortex, Th: thalamus). Self-blocking by co-administration of 10 nM istradefylline (B). The binding is completely blocked by co-administration of 10 µM of the A_{2A} receptor antagonist ZM241385 (C). Representative competition curve (D). 15

Fig. S5: Representative *in vitro* autoradiographic images of the binding pattern of [^{18}F]**FLUDA** in horizontal mouse brain slices. The highest accumulation of activity in the striatum (A, red; St: striatum, Cb: cerebellum, Cx: cortex, Th: thalamus). Self-blocking by co-administration of 10 nM tozadenant (B). The binding is completely blocked by co-administration of 10 µM of the A_{2A} receptor antagonist ZM241385 (C). Representative competition curve (D). 15

Fig. S6: *In vitro* autoradiographic images of the binding pattern of [^{18}F]**FLUDA** in sagittal pig brain slices. The highest accumulation of activity is in the striatum (A, red, St: striatum, Cb: cerebellum, Cx: cortex, Th: thalamus). Self-blocking by co-administration of 10 nM istradefylline (□). The binding is completely blocked by co-administration of 10 µM of the A_{2A} receptor antagonist ZM241385 (C). Competition curve (D). 16

Fig. S7: *In vitro* autoradiographic images of the binding pattern of [^{18}F]**FLUDA** in sagittal pig brain slices. The highest accumulation of activity is in the striatum (A, red, St: striatum, Cb: cerebellum, Cx: cortex, Th: thalamus). Self-blocking by co-administration of 10 nM tozadenant (B). The binding is completely blocked by co-administration of 10 µM of the A_{2A} receptor antagonist ZM241385 (C). Competition curve (D). 16

Fig. S8: Area under the curve of $SUVr_{St/Cb}$ over time after pre-treatment with vehicle (red square, n = 8), tozadenant (2.5 mg/kg bw, blue circle, n = 4) and istradefylline (1.0 mg/kg bw, green triangle, n = 4). Student T-test: $p < 0.05$* 17

Tab. S2: Tissue biodistribution of radioactivity at different time point after intravenous injection of [^{18}F]**FLUDA** in CD-1 mice based on PET imaging. 17

Fig. S9: (A) Biodistribution of [^{18}F]**FLUDA** in CD-1 mice at different time points based on PET imaging (n = 4); Representative horizontal merged PET/MRI images of [^{18}F]**FLUDA** biodistribution focused on (B) kidneys and liver and (C) small intestine and bladder, and (D) corresponding maximal intensity projection (MIP). K: kidneys; L: liver; SI: small intestine; B: bladder. 18

References 19

Material and methods

General – All chemicals and reagents were purchased from commercially available sources and used without further purification. The A_{2A} receptor antagonists **tozadenant** (SYN-115; K_i(hA_{2A} receptor) = 11.5 nM [1]) and **istradefylline** (KW-6002; K_i(A_{2A} receptor) = 12 nM [2]) were obtained from abcr GmbH (Karlsruhe; Germany) and Bio-Tehne GmbH (Wiesbaden-Nordenstadt; Germany), respectively. Microwave-assisted reactions were performed with a CEM Discover LabMate Microwave. The purity was determined by LC-MS [Dionex Ultimate 3000 system incorporating a LPG-3400SD pump, a WPS-3000 TSL autosampler, a TCC-3000SD column compartment, a DAD3000 diode array detector and a MSQ 3000 low resolution mass spectrometer (Thermo Fisher Scientific Inc.; Waltham; USA), column: Reprosil-Pur Basic HD (150×3 mm; 3 µm; Dr. Maisch GmbH; Ammerbuch; Germany), gradient: 10-90-10% MeCN/20 mM $NH_4OAc_{aq.}$ (*v/v*, 15 min, 0.6 mL/min]. ^{1}H-, ^{13}C- and ^{19}F-NMR spectra were recorded on a BRUKER DRX-400 (400 MHz for ^{1}H-NMR, 100 MHz for ^{13}C-NMR, 377 MHz for ^{19}F-NMR). All spectra were recorded at room temperature followed by calibration on the solvent signal [DMSO-d_6: δ(^{1}H-NMR) = 2.50 ppm and δ(^{13}C-NMR) = 39.52 ppm]. Multiplicities of NMR signals are indicated as follows: s (singlet), d (doublet), t (triplet), q (quartet), p (pentet), m (multiplet), dd (doublet of doublets). High resolution mass spectra (HRFT-MS) were recorded on a BRUKER FT-ICR APEX II spectrometer using electrospray ionization (ESI). Analytical thin-layer chromatography (TLC) was performed on silica gel coated plates (Macherey-Nagel, ALUGRAM SIL G/UV254). The spots were detected by using an UV lamp or by dipping the plates into a $KMnO_4$-solution (3 g $KMnO_4$, 20 g K_2CO_3, 0.25 mL glacial acid, 300 mL water). Purification of the final products was assessed by flash column chromatography with silica gel 40–63 µm from VWR Chemicals.

Chemical synthesis – Ethane-1,2-diyl-d_4 bis(4-methylbenzenesulfonate) (**2**), 2-fluoroethyl-1,1,2,2-d_4 4-methylbenzenesulfonate (**3**) and 4-[3-(5-amino-2-furan-2-yl-pyrazolo[4,3-*e*][1,2,4]triazolo[1,5-*c*]pyrimidin-7-yl)-propyl]-phenol (**desmethyl SCH442416**) were synthesized as described by literature.[3-7]

7-(3-(4-(2-Fluoroethoxy-1,1,2,2-d_4)phenyl)propyl)-2-(furan-2-yl)-7*H*-pyrazolo-[4,3-*e*][1,2,4]triazolo[1,5-*c*]pyrimidin-5-amine (FLUDA): The synthesis of **FLUDA** was performed by a microwave-assisted reaction of **desmethyl SCH442416** (50.7 mg, 1 eq, 0.14 mmol) and 2-fluoroethyl-1,1,2,2-d_4 4-methylbenzenesulfonate (**3**, 90 mg, 3 eq, 0.41 mmol) with cesium carbonate (132 mg, 3 eq, 0.41 mmol) in methanol (2.5 mL) at 100 °C/100 W for 1 h. After removal of the solvent by rotatory evaporation, the remaining residue was dissolved in ethyl acetate (50 mL) and the organic phase was washed with water (3 x 50 mL) and brine (20 mL), dried over anhydrous magnesium sulfate, filtered and evaporated to dryness. The crude product was purified twice by flash chromatography (silica, 1st gradient dichloromethane/methanol 100:1→100:2→100:3 and 2nd gradient ethyl acetate/petroleum ether 3:1→ 4:1→5:1 to give **FLUDA** (21 mg, 0.05 mmol, 37%) as tan solid. TLC (silica gel, CH_2Cl_2/MeOH,

9:1): R_f = 0.71; LC-MS: t_R = 11.1 min, > 97%; ^{1}H-NMR (400 MHz, DMSO-d_6): δ = 8.16 (s, 1H), 8.06 (s, NH_2), 7.94 (dd, J = 0.9, 1.8 Hz, 1H), 7.23 (dd, J = 0.8, 3.4 Hz, 1H), 7.13 (d, J = 8.7 Hz, 2H), 6.86 (d, J = 8.6 Hz, 2H), 6.73 (dd, J = 1.8, 3.3 Hz, 1H), 4.25 (t, J = 6.9 Hz, 2H), 2.54 (t, J = 7.6 Hz, 2H), 2.11 (p, J = 7.2 Hz, 2H); ^{19}F-NMR (376 MHz, DMSO-d_6) δ = -223.94; ^{13}C-NMR (101 MHz, DMSO-d_6): δ = 156.87, 155.81, 149.19, 148.86, 146.73, 146.00, 145.55, 133.80, 131.77, 129.84 (2C), 114.83 (2C), 112.72, 112.59, 96.20, 83.70–79.95 (m), 67.71–65.26 (m), 46.54, 31.75, 31.38; HRFT-MS (ESI+): m/z = 448.18060 (calcd. 448.18058 for $[M+Na]^+$).

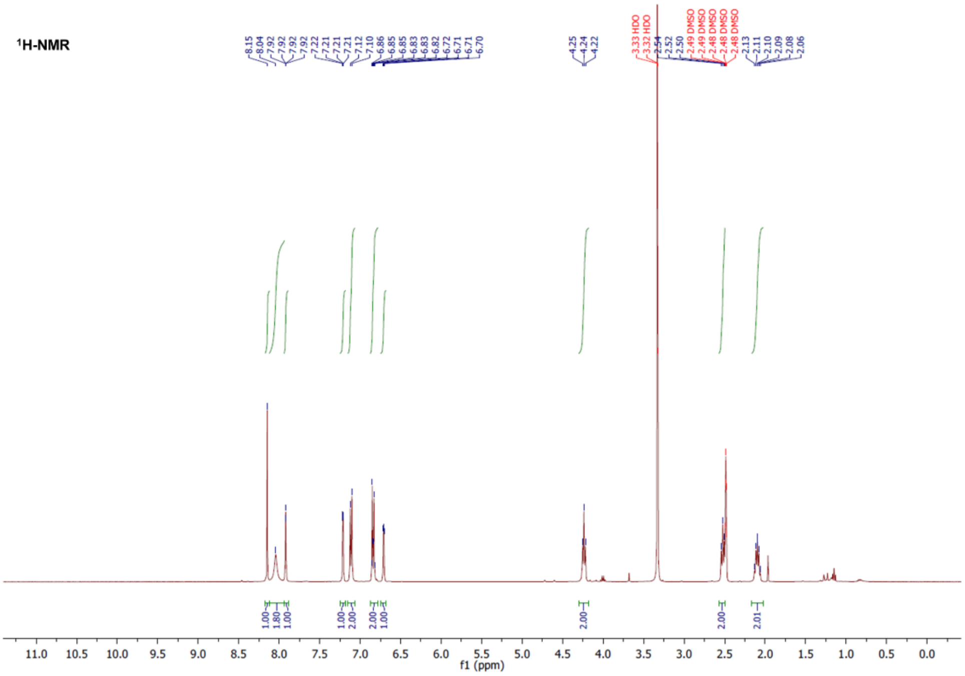

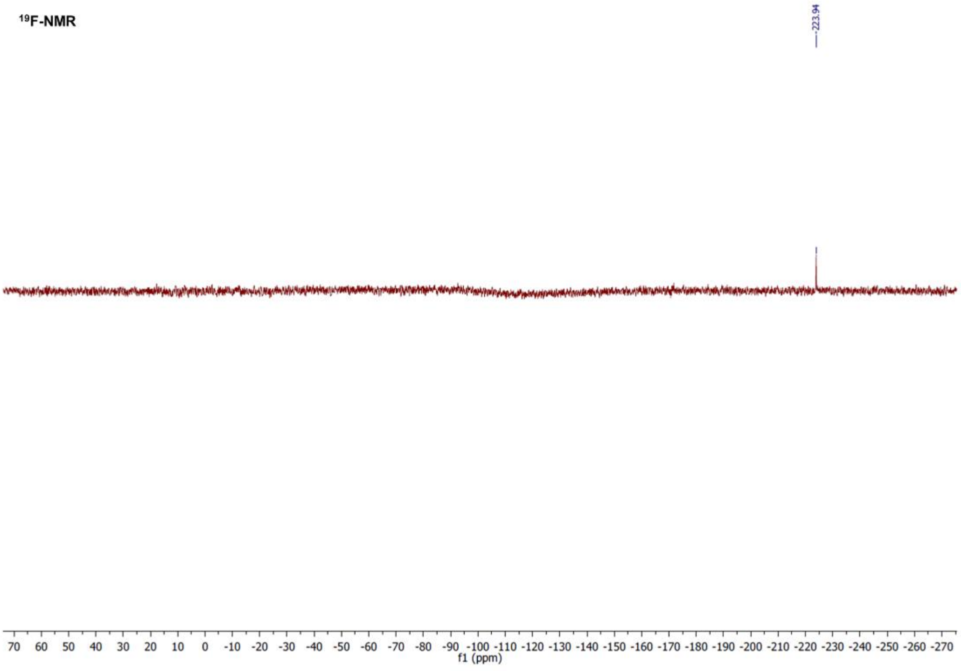

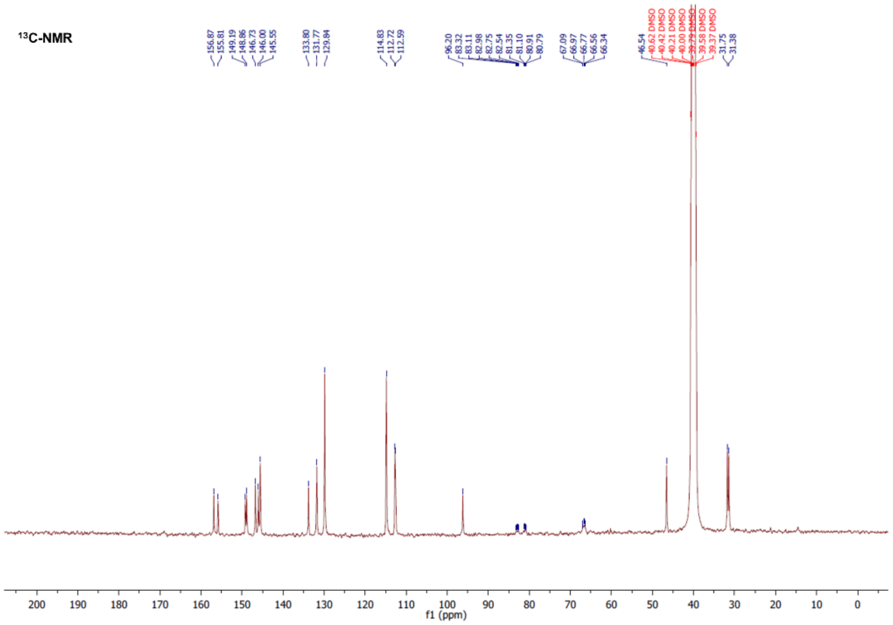

Radiosynthesis of [^{18}F]FLUDA: No carrier added (n.c.a.) [^{18}F]fluoride was produced *via* the [^{18}O(p,n)^{18}F] nuclear reaction by irradiation of an [^{18}O]H_2O target (Hyox 18 enriched water; Rotem Industries Ltd; Mishor Yamin; Israel) on a Cyclone 18/9 Cyclotron (iba RadioPharma Solutions; Louvain-la-Neuve; Belgium) with fixed energy proton beam using Nirta [^{18}F]fluoride XL target. N.c.a. [^{18}F]fluoride in 1.0 mL of water was trapped on a pre-conditioned Sep-Pak Accell Plus QMA Carbonate Plus light cartridge (Waters GmbH; Eschborn; Germany, pre-conditioned with 15 mL 0.5 M $NaHCO_3$ and 10 mL H_2O). Then, the activity was eluted with 300 µL of an aqueous potassium carbonate solution (1.78 mg, 12.9 µmol) in a conical 4 mL vial with a solution kryptofix® (K_{222}, 5.6 mg, 14.9 µmol) in 1 mL of MeCN. The aqueous [^{18}F]fluoride was azeotropically dried under vacuum and nitrogen flow within 7-10 min using a CEM Discover PETwave Microwave (CEM GmbH; Kamp-Lintfort; Germany; 75 W, at 50-60 °C, power cycling mode). Two aliquots of anhydrous MeCN (2x1.0 mL) were added during the drying procedure and then, the final K[^{18}F]F-K_{222} complex was dissolved in 400 µL MeCN. Then, the K[^{18}F]F-K_{222} complex was stirred with tosylate precursor **2** (3 mg) in 100 µL MeCN at 90 °C for 10 min. In parallel the **desmethyl SCH4424167** precursor (3 mg) was stirred with 490 µL MeCN and 10 µL TBAOH (40 wt.% in H_2O) at 90 °C for 10 min. Thereafter, the pre-treated **desmethyl SCH442416** precursor was added to the non-isolated 2-[^{18}F]fluoroethyl-1,1,2,2-d_4 4-methylbenzenesulfonate ([^{18}F]**3**) and the reaction mixture was heated at 120 °C for 10 min. After cooling, the reaction mixture was diluted with 1 mL H_2O and applied directly onto a RP-HPLC column (ReproSil-Pur 120 C18-AQ column: 250×20 mm, 5 µm; Dr. Maisch HPLC GmbH; Ammerbuch; Germany) using a mixture of 58% MeCN/20 mM $NH_4OAc_{aq.}$ (*v/v*) as eluent and a flow rate of 7 mL/min (R_t ~ 21 min). The fractions containing [^{18}F]**FLUDA** were collected and diluted with water to a total volume of 40 mL. Thereafter, the solution was passed through a pre-conditioned Sep-Pak® C18 Plus cartridge (Waters GmbH; Eschborn; Germany; pre-conditioned with 5 mL EtOH and 60 mL H_2O),

washed with 2 mL water, and [^{18}F]**FLUDA** was subsequently eluted with 1.5 mL absolute ethanol. After the addition of 30 µL DMSO, the solvent was reduced under a stream of nitrogen at 70 °C (approx. 50 µL), and [^{18}F]**FLUDA** was formulated with the addition of sterile isotonic saline solution up to a final concentration of $\leq$ 10% EtOH (*v/v*) and $\leq$ 10% DMSO (*v/v*). Radiochemical and chemical purities were assessed by radio-TLC and analytical HPLC. Molar activities were determined based on aliquots taken from the formulation, and the mass determination for the corresponding reference standard was performed *via* a calibration curve (0.02 – 6 µg **FLUDA**) obtained under the same analytical HPLC conditions (see quality control).

Quality control – Radio-TLC was performed on silica gel pre-coated plates (Polygram® SIL G/UV254; Roth; Germany) with a mixture of ethyl acetate/petroleum ether 6/1 (*v/v*) as eluent. The plates were exposed to storage phosphor screens (BAS-IP MS 2025; FUJIFILM Co.; Japan) and recorded using the Amersham Typhoon RGB Biomolecular Imager (GE Healthcare Life Sciences). Images were quantified with the ImageQuant TL8.1 software (GE Healthcare Life Sciences). Analytical radio-HPLC was performed on a JASCO LC-2000 system, incorporating a PU-2080Plus pump, AS-2055Plus auto injector (100 µL sample loop), and a UV-2070Plus detector coupled with a gamma detector (Gabi Star; raytest Isotopenmessgeräte GmbH; Germany). Data analysis was performed with the Galaxie chromatography software (Agilent Technologies) using the chromatograms obtained at 254 nm. An analytical Reprosil-Pur C18-AQ column (250×4.6 mm; particle size: 5 µm) and a semi-preparative Reprosil-Pur C18-AQ column (150×10 mm; particle size: 10 µm) from Dr. Maisch HPLC GmbH (Ammerbruch; Germany) were used with an eluent mixture of MeCN/20 mM $NH_4OAc_{aq.}$ (pH 6.8) in gradient mode (0–5 min 18% MeCN, 5–20 min up to 90% MeCN, 20–22 min 90% MeCN, 22–23 min up to 10% MeCN, 23–30 min 10% MeCN) at a flow rate of 1 or 3 mL/min, respectively. The ammonium acetate concentrations stated as 20 mM $NH_4OAc_{aq.}$ correspond to the concentration in the aqueous component of an eluent mixture. The molar activities were determined on the basis of a calibration curve carried out under isocratic conditions (36% MeCN/20 mM $NH_4OAc_{aq.}$; analytical Reprosil-Pur C18-AQ column) using chromatograms obtained at 264 nm as the maximum of UV absorbance.

Physiochemical properties – The *in vitro* stability of [^{18}F]**FLUDA** was investigated by incubation of approx. 10 MBq at 37 °C in 500 µL NaCl, PBS, *n*-octanol and pig plasma samples. After 15, 30 and 60 min aliquots were taken and analysed by radio-TLC and radio-HPLC. The partition coefficient of [^{18}F]**FLUDA** was experimentally determined for the *n*-octanol/PBS system by the shake-flask method. Approx. 900 kBq [^{18}F]**FLUDA** were added to a mixture of 3 mL of *n*-octanol and 3 mL of PBS. After shaking for 20 min at room temperature (RT), the samples were centrifuged (10,000 rpm; 5 min) and 1 mL aliquots of the organic as well as the aqueous layer were taken and measured in a gamma counter (Wizard 1480; Perkin Elmer; Turku; Finland). Another 1 mL aliquot of the organic layer was mixed

with 2 mL *n*-octanol and 3 mL of PBS and subjected to the same procedure until constant partition coefficient values had been obtained. The measurement was performed in quadruplicate.

Biological evaluation – All animal studies followed the international guidelines of animal care and were approved by Landesdirektion Leipzig, the local authority for animal care (Reg.-Nr.: TVV 18/18; Reference number DD24.1-5131/446/19). The animal experiments were performed with female CD-1 mice (n = 23, 10-12 weeks, 26-38 g) obtained from the Medizinisch-Experimentelles Zentrum (MEZ) at Universität Leipzig (Leipzig; Germany) and female piglets (n = 2, 6-12 weeks old, 14-18 kg) obtained from the Lehr- und Versuchsgut of the Faculty of Veterinary Medicine at Universität Leipzig (LVG; Oberholz; Germany).

***In vitro* binding assays –** Binding assays were performed as previously described.[8] CHO-K1 cells stably transfected with the human A_1 receptor or A_{2A} receptor, were donated by Prof. Karl-Norbert Klotz (Institute of Pharmacology and Toxicology, Universität Würzburg; Würzburg; Germany) were cultured in DMEM/F12 medium supplemented with 15 mM HEPES, 10% FCS, 1% L-Glutamine, 1% Penicillin/Streptomycin, and G418 as selective antibody at 0.2 mg/mL at 5% CO_2 and 37 °C. Cells were harvested by scraping followed by centrifugation (800 rpm, 5 min), and the resulting pellet incubated in 50 mM TRIS-HCl buffer (pH 7.4, 100 mM NaCl, 5 mM $MgCl_2$, 1 mM EDTA) on ice for 20 min. The crude membrane homogenate was obtained by centrifugation (15,000 rpm, 30 min, 4 °C), suspended in 50 mM TRIS-HCl and stored at –25 °C. The following radiotracers were employed: [^{3}H]**ZM241385** for the A_{2A} receptor and [^{3}H]**DPCPX** for the A_1 receptor. For the determination of A_{2A} receptor and A_1 receptor binding affinity of reference compounds, frozen cell suspensions were thawed, homogenized by a 27-gauge needle, and diluted with 50 mM TRIS-HCl buffer containing 1 µU/mL adenosine deaminase (ADA). Membrane suspension was incubated with 1.5 nM [^{3}H]**ZM241385** or 1 nM [^{3}H]**DPCPX** and various concentrations of **FLUDA**. Non-specific binding was determined by co-incubation with 10 µM **ZM241385** or 1 µM **DPCPX**. The incubation was performed at RT for 90 min and terminated by rapid filtration using Whatman GF/B glass-fibre filters, presoaked in 0.3% polyethyleneimine, and a 48-channel harvester (Biomedical Research and Development Laboratories; Gaithersburg; MD; USA), followed by washing four times with ice-cold TRIS-HCl buffer. Filter-bound radioactivity was quantified by liquid scintillation counting. The K_i values were obtained by different concentrations (n≥7) in triplicate. The IC_{50} values were determined by non-linear regression analysis with GraphPad Prism 4.1 (GraphPad Inc.; La Jolla; CA), and *K*i values estimated according to the Cheng-Prusoff equation with $K_{D,ZM241385}$(*h*A_{2A} receptor) = 0.8 nM and $K_{D,DPCPX}$(*h*A_1 receptor) = 0.45 nM.

***In vitro* autoradiography** – Binding studies on brain sections were performed as previously described.[9] Brains of CD-1 mice frozen in isopentane were cut using a cryostat (MICROM HM560; Fisher Scientific GmbH; Schwerte; Germany), thaw-mounted onto microscope slides, and after air-

drying stored at - 80 °C until use. The brain sections were dried in a stream of cold air, and pre-incubated in 50 mM TRIS-HCl buffer (pH 7.4, 100 mM NaCl, 5 mM $MgCl_2$, 1 mM EDTA) containing 1 µU/mL adenosine deaminase (ADA) for 15 min at RT. Afterwards, brain sections were incubated with approx.. 1 MBq/mL of [^{18}F]**FLUDA** in buffer for 90 min at room temperature. Non-specific binding was determined in the presence of 10 µM **ZM241385**. Displacement of [^{18}F]**FLUDA** was also evaluated with 10 µM **FLUDA**, **tozadenant** and **istradefylline**. Subsequently, the sections were washed twice for 5 min in ice-cold TRIS-HCl buffer, and dipped for 5 s in ice-cold deionized water. The sections were rapidly dried in a stream of cold air before being exposed overnight on an imaging plate. Developed autoradiographs were analysed in a phosphor imager (HD-CR 35; Duerr NDT GmbH; Bietigheim-Bissingen; Germany). The quantification was performed by using 2D-densitometric analysis (AIDA 2.31 software; raytest Isotopenmessgeräte GmbH; Straubenhardt; Germany). Further data analysis was performed with GraphPad Prism 4.1 (GraphPad Inc.; La Jolla; CA).

***In vivo* metabolism in mice –** Stability studies of [^{18}F]**FLUDA** in mice were performed as previously described.[9] The radiotracer was intravenously (i.v.) administered as bolus in awake CD-1 mice (~ 36 MBq [^{18}F]**FLUDA**; n = 3). Brain and blood samples were obtained at 15 min post injection (p.i.), plasma was separated by centrifugation at 12,000 rpm at RT for 1 min (Centrifuge 5418; Eppendorf Vertrieb Deutschland GmbH; Wesseling-Berzdorf; Germany), and brain homogenized in 1 mL water on ice (10 strokes of a PTFE plunge at 1000 rpm in a borosilicate glass cylinder; Potter S Homogenizer; B. Braun Melsungen AG; Melsungen; Germany). All samples were weighed and the respective activity measured in a dose calibrator (ISOMED 2010; MED Nuklear-Medizintechnik Dresden GmbH; Dresden; Germany).

RR-HPLC: For protein precipitation and extraction an ice-cold mixture of acetone/water (4/1; *v/v*) was used in a ratio of 4:1 (*v/v*) of solvent to plasma or brain homogenate, respectively. The samples were vortexed for 3 min, equilibrated on ice for 5 min, and centrifuged for 5 min at 10,000 rpm. After separating the supernatant, the precipitates were washed with 100 µL of the solvent mixture and subjected to the same procedure. The combined supernatants were concentrated at 75 °C under nitrogen flow to a final volume of approximately 100 µL and analysed by analytical radio-HPLC. Separations were performed by using a Reprosil-Pur C18-AQ column (250×4.6 mm; 5 µm; Dr. Maisch HPLC GmbH; Ammerbuch; Germany) and an eluent mixture of MeCN/20 mM $NH_4OAc_{aq.}$ (pH 6.8) in gradient mode (0–5' 18% MeCN, 5–20' up to 90% MeCN, 20–22' 90% MeCN, 22–23' up to 10% MeCN, 23–30 min 10% MeCN) at a flow rate of 1.0 mL/min. To determine the percentage of radioactivity in the supernatants compared to total activity, aliquots of each step as well as the precipitates were quantified by a gamma counter (Wallac Wizard 1480; Perkin Elmer; Turku; Finland). With this procedure, recoveries of about 84-98% for plasma samples and brain homogenates could be obtained.

MLC: For preparation of the MLC injection samples, mouse plasma (30 µL, 15 min p.i.) was dissolved in 170 µL of 100 mM aqueous sodium dodecyl sulfate ($SDS_{aq.}$). Homogenized brain material (300 µL,

15 min p.i.) was dissolved in 400 µL of 200 mM $SDS_{aq.}$, stirred at 70 °C for 5 min, diluted with more 200 µL of 200 mM $SDS_{aq.}$, centrifuged for 5 min at 10,000 rpm and injected into the MLC system. The MLC system was built up of a JASCO PU-980 pump, an AS-2055Plus auto injector with a 2 mL sample loop, and a UV-1575 detector coupled with a gamma radioactivity HPLC detector (Gabi Star, raytest Isotopenmessgeräte GmbH; Straubenhardt; Germany). Data analysis was performed with the Galaxie chromatography software (Agilent Technologies). Separations were performed by using a Reprosil-Pur 200 C18-AQ column (250×4.6 mm, 10 µm; Dr. Maisch HPLC GmbH; Ammerbuch; Germany) and an eluent mixture of EtOH/100 mM $SDS_{aq.}$/25 mM $(NH_4)_2HPO_{4,aq.}$ in gradient mode (brain: 0–10' 0% EtOH, 10–15' up to 10% EtOH, 15–20' 10% EtOH, 20–21' up to 0% EtOH; 21–30' 0% EtOH//100 mM $SDS_{aq.}$/25 mM $(NH_4)_2HPO_{4,aq}$ and plasma: 0–10' 0% EtOH, 10–20' up to 10% EtOH, 20–23' 10% EtOH, 23–24' up to 0% EtOH; 24–35' 0% EtOH//100 mM $SDS_{aq.}$/25 mM $(NH_4)_2HPO_{4,aq.}$) at a flow rate of 1.0 mL/min.

***In vivo* metabolism in piglets –** Stability studies of [^{18}F]**FLUDA** in piglets were performed as previously described [11, 12]. Two piglets were fasted on the day of imaging and received an intramuscular injection of 1 mL azaperone and 4 mL ketamine to introduce anaesthesia. After 15 min, 2 mL of ketamine and 1 mL of midazolam (5 mg/mL) were i.v. injected (ear vein, V. auricularis), followed by 5 mL of G40, 3 mL of ketamine, and 1.5 mL of midazolam in 50 mL of NaCl 0.9% with an infusion pump at a flow rate of 37.5 mL/h to maintain the narcosis throughout the entire investigation time. During narcosis, the animals maintained spontaneous respiration and no mandatory ventilation was applied. The radiotracer was injected as a bolus (5 mL saline with ~ 203 MBq [^{18}F]**FLUDA**, n = 2) into the auricular vein of anesthetized piglets. Arterial blood was sampled over 120 min and the blood plasma was obtained by centrifugation at 12,000 rpm at RT for 1 min (Centrifuge 5418; Eppendorf Vertrieb Deutschland GmbH; Wesseling-Berzdorf; Germany). For protein precipitation and extraction an ice-cold mixture of acetone/water (4/1; *v/v*) was used in a ratio of 2:1 (*v/v*) of solvent (4 mL) to plasma (2 mL). The samples were vortexed for 3 min, equilibrated on ice for 5 min, and centrifuged for 5 min at 5,000 rpm. The combined supernatants were concentrated at 75 °C under nitrogen flow to a final volume of approximately 1-3 mL and analysed by semi-preparative radio-HPLC. Separations were performed by using a Reprosil-Pur C18-AQ column (150×10 mm, 10 µm; Dr. Maisch HPLC GmbH; Ammerbuch; Germany) and an eluent mixture of MeCN/20 mM NH_4OAc_{aq}. (pH 6.8) in gradient mode (0-5' 18% MeCN, 5-20' up to 90% MeCN, 20-22' 90% MeCN, 22-23' up to 18% MeCN, 23-30' 18% MeCN) at a flow rate of 3.0 mL/min. To determine the percentage of radioactivity in the supernatants compared to total activity, aliquots of each step as well as the precipitates were quantified by a gamma counter (Wallac Wizard 1480; Perkin Elmer; Turku; Finland). With this procedure, recoveries of about 82 - 90% were obtained for plasma samples.

Dynamic PET studies in mice – PET imaging was performed according to the protocol previously described.[13] For the time of the experiments, CD-1 mice (n = 20) were kept in a dedicated climatic chamber with free access to water and food under a 12:12 h dark:light cycle at a constant temperature (24 °C). The animals were anaesthetized (anaesthesia unit U-410; agntho's; Lidingö; Sweden) with isoflurane (1.8%, 0.35 L/min) delivered in a 60% oxygen / 40% air mixture (Gas Blender 100 Series; MCQ instruments; Rome; Italy) and maintained at 37 °C with a thermal bed system. [^{18}F]**FLUDA** was injected into the tail vein (3.1-9.7 MBq in 150 µL isotonic saline; A_m: 72-376 GBq/µmol, EOS; 0.7 - 4.5 nmol/kg) followed by a 60 min PET/MR scan (PET/MR 1Tesla; nanoScan®; MEDISO Medical Imaging Systems; Budapest; Hungary): 4 baseline studies, 8 control studies with vehicle (DMSO/Kolliphor/NaCl, 1:2:7, v/v/v), 4 pre-treatment studies with **tozadenant** (2.5 mg/kg; abcr GmbH; Karlsruhe; Germany) and 4 pre-treatment studies with **istradefylline** (1.0 mg/kg; Bio-Tehne GmbH; Wiesbaden-Nordenstadt; Germany). Vehicle and blocking agents were administrated 15 min and 8 min prior radiotracer injection for **tozadenant** and **istradefylline** respectively. Each PET image was corrected for random coincidences, dead time, scatter and attenuation (AC), based on a whole body (WB) MR scan. The list mode data were sorted into sonograms using a framing scheme of 12x10 s, 6x30 s, 5x300 s, 9x600 s. The reconstruction parameters for the list mode data were 3D-ordered subset expectation maximization (OSEM), 4 iterations, 6 subsets, energy window: 400-600 keV, coincidence mode: 1-5, ring difference: 81. The mice were positioned prone in a special mouse bed (heated up to 37 °C), with the head fixed to a mouth piece for the anaesthetic gas supply with isoflurane in 40% air and 60% oxygen (anaesthesia unit: U-410, agnthos, Lidingö, Sweden; Gas blender: MCQ, Rome, Italy). The PET data was collected by a continuous WB scan during the entire investigation. Following the 60 min PET scan a T1 weighted WB gradient echo sequence (TR/TE: 20/6.4 ms, NEX: 1, FA: 25, FOV: 64x64 mm, matrix: 128x128, slice thickness: 0.5 mm) was performed for AC and anatomical orientation. Image registration and evaluation of the volume of interest (VOI) was done with PMOD v3.9 (PMOD technologies LLC; Zurich; Switzerland). The respective brain regions were identified using the mouse brain atlas template Ma-Benveniste-Mirrione-FDG. The blood compartment was delineated based on the PET first time frame showing the vena cava. The small intestine was delineated based on the PET image, and liver, heart, spleen, kidney and muscle were manually delineated based on the T1-weighted MRI inside the boundary of the organ to avoid spill-over effect. The activity data is expressed as mean standardized uptake value (SUV) of the overall ROI. Data are presented as mean ± standard deviation (SD). Microsoft Office Excel (2016) was used to perform statistical tests. A parametric student T-test preceded by a Fischer test for variance was used to compare the groups with $p < 0.05$.

PET studies in piglets – PET imaging was performed according to the protocol previously described [14]. Two piglets were fasted on the day of imaging and received an intramuscular injection of 1 mL azaperone and 4 mL ketamine to introduce anaesthesia. After 15 min, 2 mL of ketamine and 1 mL of midazolam (5 mg/mL) were i.v. injected (ear vein, V. auricularis), followed by 5 mL of G40, 3

mL of ketamine, and 1.5 mL of midazolam in 50 mL of NaCl 0.9% with an infusion pump at a flow rate of 37.5 mL/h to maintain the narcosis throughout the entire investigation time. During narcosis, the animals maintained spontaneous respiration and no mandatory ventilation was applied. Dynamic PET scans of a control study and a pre-treatment study with administration of 2.5 mg/kg **tozadenant** 15 min prior to radiotracer injection followed by a continuous infusion throughout the scan (0.9 mg/kg/h) were acquired during 90 min after an i.v. injection (contralateral ear vein) of 178 - 229 MBq [^{18}F]**FLUDA** (0.08 - 0.16 fmol/kg, n = 2) in a stand-alone PET system (ECAT Exact HR^{+}, SIEMENS; Erlangen; Germany). The piglets were positioned prone with legs alongside the body on a custom-made plastic trough including a piglet head-holder. The dynamic PET acquisition (23 frames: 4 x 15 sec, 4 x 60 sec, 5 x 120sec, 5 x 300 sec, 5 x 600 sec) was carried out following a cold transmission scan using Ge^{68}-line sources for attenuation correction (AC) of the PET emission data. PET data was reconstructed into a 128 x 128 x 63 matrix (0.257 x 0.257 x 0.243 cm^3 voxel size) involving AC and employing an iterative OSEM algorithm with 10 iterations and 16 subsets. The reconstructed data was processed using PMOD v3.9 (PMOD technologies LLC; Zurich; Switzerland).

Toxicity studies in rats – The extended single dose toxicity studies of **FLUDA** in male (n = 45) and female (n = 45) outbreed Wistar rats were performed in the Biological Testing Laboratory (BTL) in Russia (study number 678/19). The test item **FLUDA** was dissolved in vehicle (35% DMSO in saline) and administered by single bolus i.v. injection at the doses 6 and 30 µg/kg body weight (bw). The maximum dose level of 30 µg/kg bw is approx. 4000-fold of an estimate human dose based to the ICH guideline M3(R2). A concurrent control group received the vehicle in the same volume of 3 mL/kg bw. Ten animals per sex/group were euthanized on the second day (24 h after administration) and the remaining animals were euthanized after a recovery period of two weeks with terminal blood collection for clinical pathology evaluations (hematology and serum chemistry) and necropsied. Selected organs were weighed, and selected tissues were examined microscopically. There was no morbidity and mortality of male and female rats caused by the single intravenous FLUDA administration at the doses of 6 and 30 µg/kg bw. A summary of the doses descriptor of systematic toxicity in presented below:

**Dose**	**Effect/Organ**
**Male**	
6 µg/kg bw	Serum chemistry: decrease in blood glucose on day 2, non-adverse, not toxicological significant
30 µg/kg bw	Serum chemistry: decrease in blood glucose on day 2, non-adverse, not toxicological significant
**Female**	
6 µg/kg bw	No significant effects
30 µg/kg bw	No significant effects

Supporting tables and figures

Tab. S1: Affinities of A_{2A} receptor antagonists in radioligand binding studies and autoradiographic studies at A_{2A} receptor.

	**mouse A_{2A} receptor**		**rat A_{2A} receptor**		**piglet A_{2A} receptor**		**human A_{2A} receptor**		
**Compound**	**K_i [a*] (nM)**	**K_D (nM)**	**K_i (nM)**	**K_D (nM)**	**K_i[a*] (nM)**	**K_D (nM)**	**K_i (nM)**	**K_D (nM)**	**References**
**Tozadenant** (SYN-115)	246 ± 84				10.8		2.04 ± 0.5[c]		this paper
							5[b]		[15]
							11.5		[1]
**Istradefylline** (KW-6002)	57.7 ± 25.5				14		33 ± 5[c]		this paper
			2.2						[16]
			13[e]						[16]
			4.46[e]						[17]
			3.8				5.7		[18]
							12		[19]
**ZM241385**	0.83 ± 0.16						0.31[c]		this paper
		0.6[*]		0.4[*]		1.8[*]			[20]
			0.3[e¶]						[21]
			1.78						[22]
							0.8[b]		[23]
**FESCH**		4.69 ± 1.17[d*]					0.6 ± 0.1[c]		[8], this paper
							12.4		[7]
**FLUDA**		4.3 ± 0.73[a*]		0.68[a*]			0.60 ± 0.02[c]		this paper

data obtained from radioligand binding studies *versus* the antagonist radioligand [^{3}H]**CGS21680**, [^{18}F]**FLUDA[a]**, [^{3}H]**SCH58261[b]**, [^{3}H]**ZM241385[c]**, [^{18}F]**FESCH[d]** or *versus* the agonist [^{3}H]**NECA[e]**
data obtained from autoradiographic studies[*]
human A_{2A} receptor affinities have been performed on membrane homogenate of CHO cells or HEK-293 cells expressing the hA_{2A} receptor recombinant protein
mouse, rat and piglet A_{2A} receptor affinities have been performed on membrane homogenate or cryosection of the striatum
radioligand binding assay performed on PC12 rat cell line

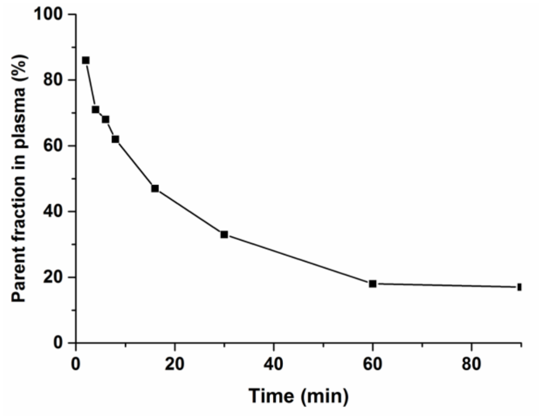

Fig. S1: Parent radiotracer fractions profile of plasma samples after administration of [^{18}F]**FLUDA** to a piglet.

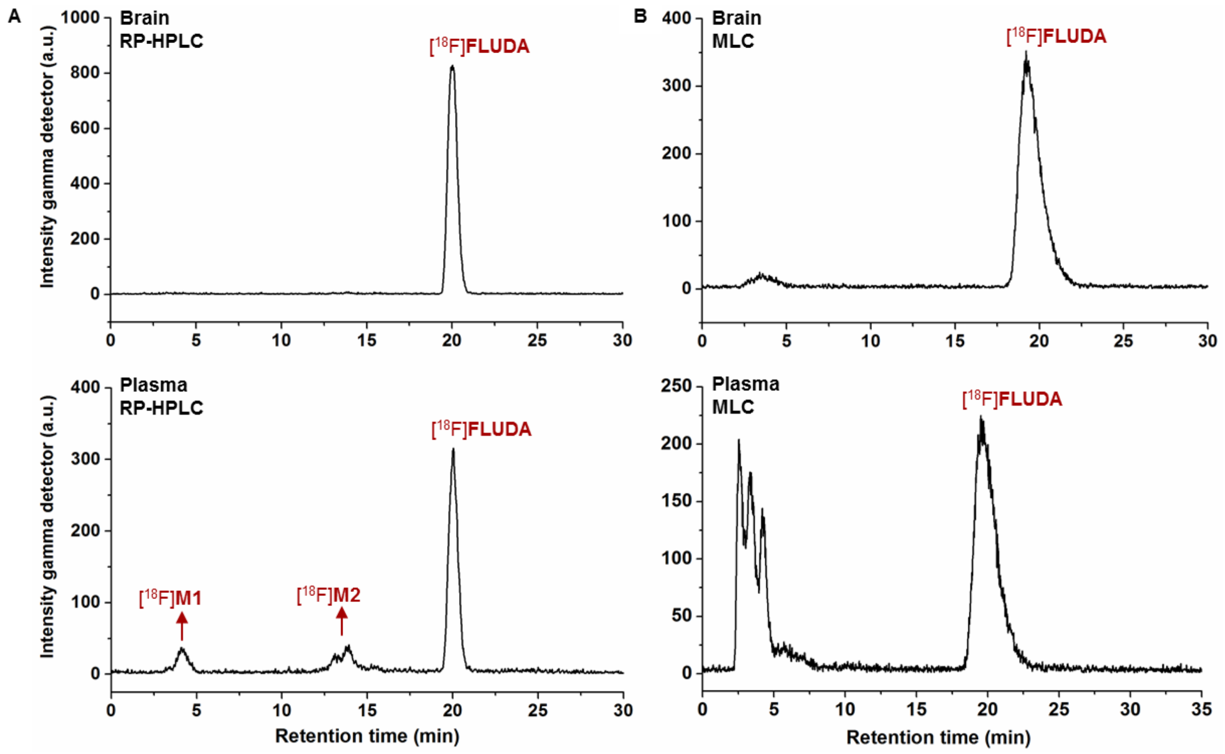

Fig. S2: Representative (**A**) RP-HPLC and (**B**) MLC radio-chromatograms of a mouse brain and plasma sample 15 min p.i. of [^{18}F]**FLUDA.**

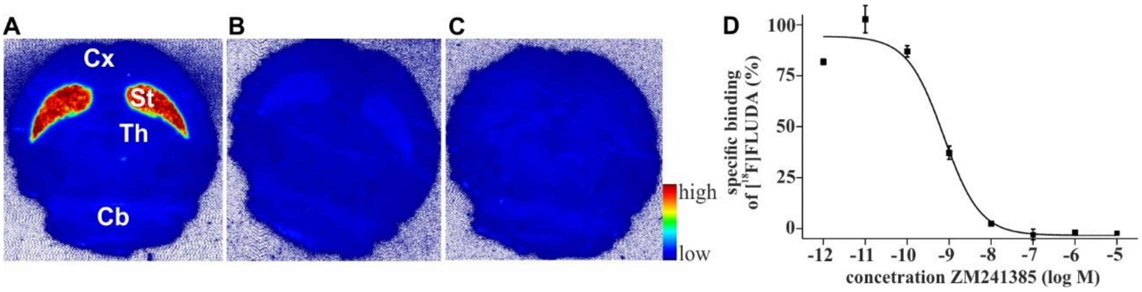

Fig. S3: Representative *in vitro* autoradiographic images of the binding pattern of [^{18}F]**FLUDA** in horizontal mouse brain slices. The highest accumulation of activity in the striatum (**A**, red; St: striatum, Cb: cerebellum, Cx: cortex, Th: thalamus). Self-blocking by co-administration of 10 nM ZM241385 (**B**). The binding is completely blocked by co-administration of 10 µM of the A_{2A} receptor antagonist istradefylline (**C**). Representative competition curve (**D**).

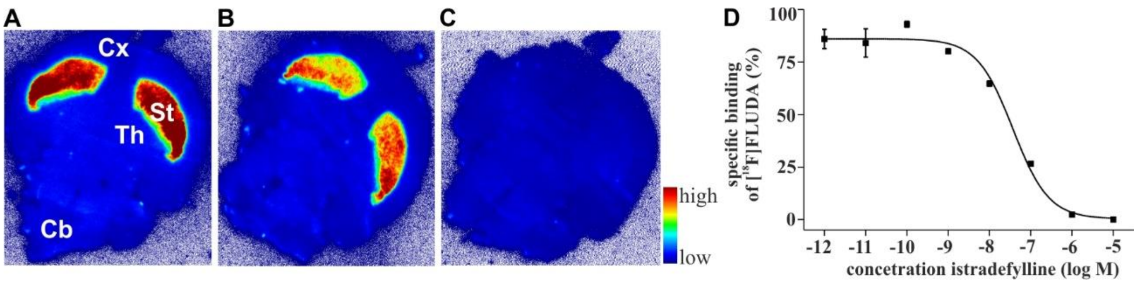

Fig. S4: Representative *in vitro* autoradiographic images of the binding pattern of [^{18}F]**FLUDA** in horizontal mouse brain slices. The highest accumulation of activity in the striatum (**A**, red; St: striatum, Cb: cerebellum, Cx: cortex, Th: thalamus). Self-blocking by co-administration of 10 nM istradefylline (**B**). The binding is completely blocked by co-administration of 10 µM of the A_{2A} receptor antagonist ZM241385 (**C**). Representative competition curve (**D**).

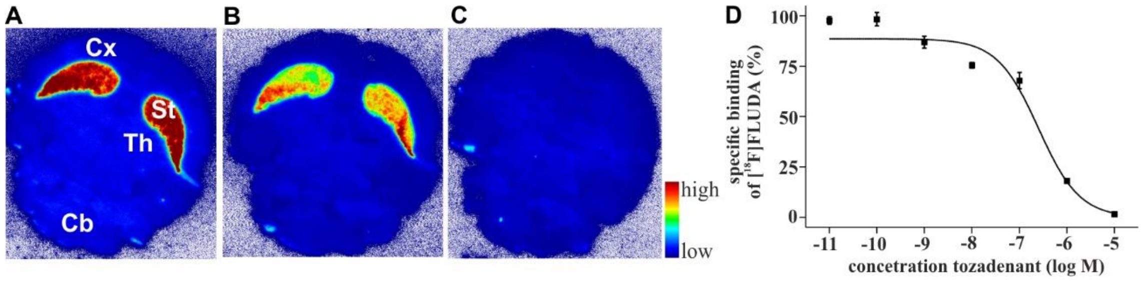

Fig. S5: Representative *in vitro* autoradiographic images of the binding pattern of [^{18}F]**FLUDA** in horizontal mouse brain slices. The highest accumulation of activity in the striatum (**A**, red; St: striatum, Cb: cerebellum, Cx: cortex, Th: thalamus). Self-blocking by co-administration of 10 nM tozadenant (**B**). The binding is completely blocked by co-administration of 10 µM of the A_{2A} receptor antagonist ZM241385 (**C**). Representative competition curve (**D**).

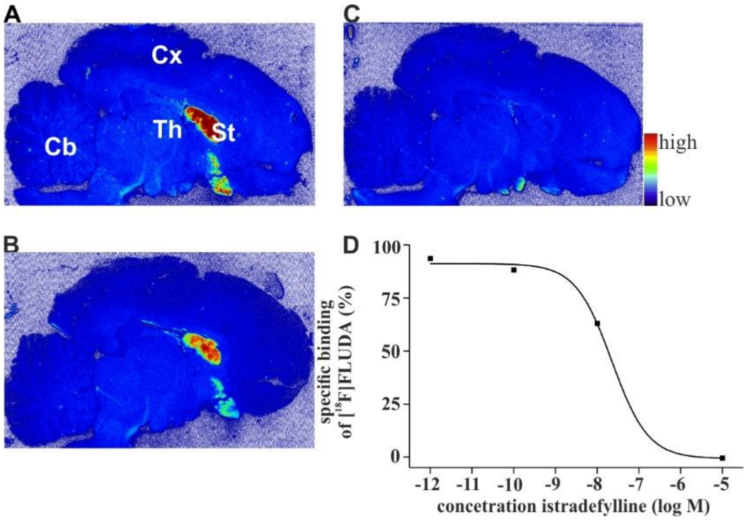

Fig. S6: *In vitro* autoradiographic images of the binding pattern of [^{18}F]**FLUDA** in sagittal pig brain slices. The highest accumulation of activity is in the striatum (**A**, red, St: striatum, Cb: cerebellum, Cx: cortex, Th: thalamus). Self-blocking by co-administration of 10 nM istradefylline (**B**). The binding is completely blocked by co-administration of 10 µM of the A_{2A} receptor antagonist ZM241385 (**C**). Competition curve (**D**).

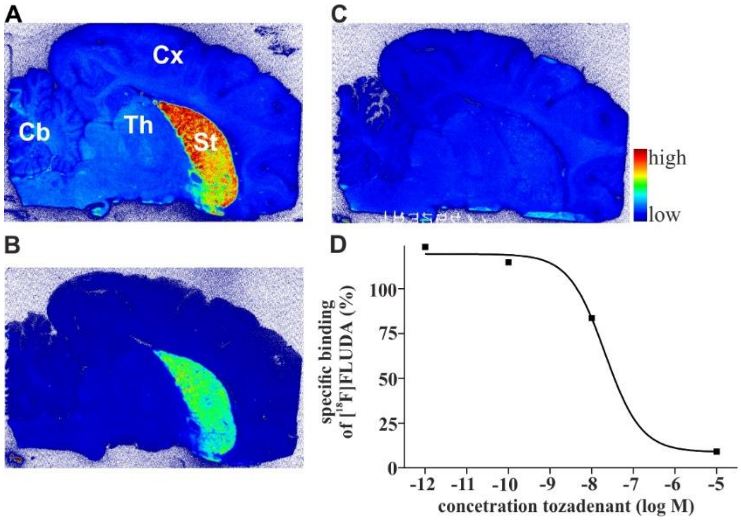

Fig. S7: *In vitro* autoradiographic images of the binding pattern of [^{18}F]**FLUDA** in sagittal pig brain slices. The highest accumulation of activity is in the striatum (**A**, red, St: striatum, Cb: cerebellum, Cx: cortex, Th: thalamus). Self-blocking by co-administration of 10 nM tozadenant (**B**). The binding is completely blocked by co-administration of 10 µM of the A_{2A} receptor antagonist ZM241385 (**C**). Competition curve (**D**).

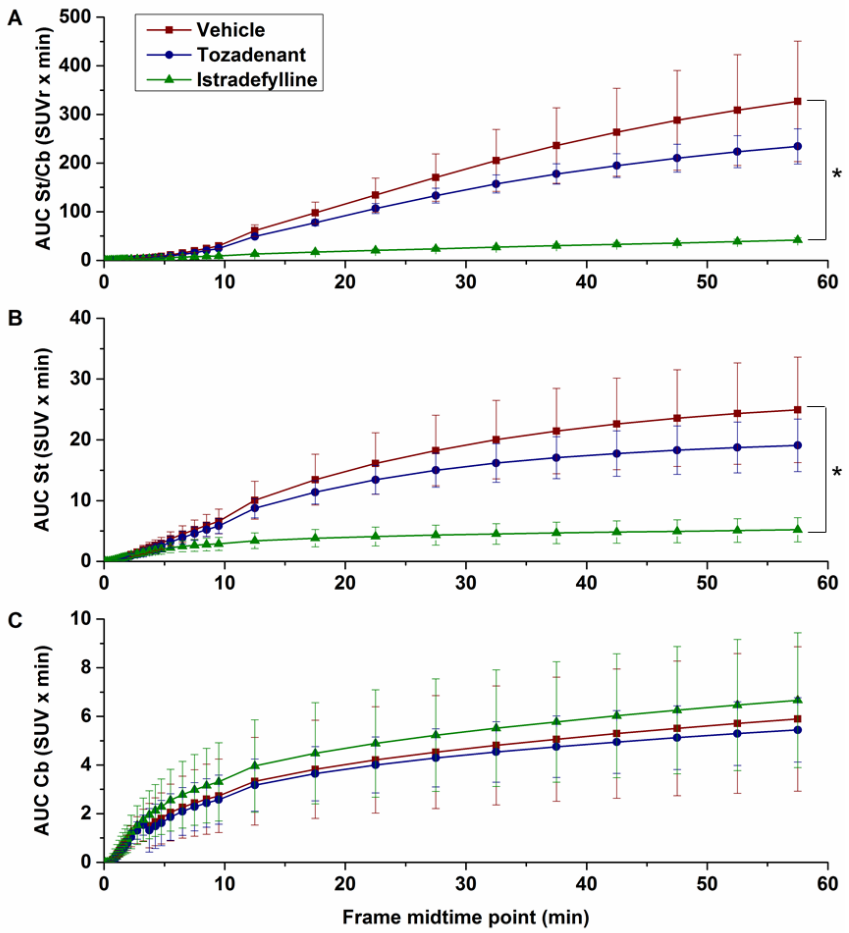

Fig. S8: Area under the curve of $SUVr_{St/Cb}$ over time after pre-treatment with vehicle (red square, n = 8), **tozadenant** (2.5 mg/kg bw, blue circle, n = 4) and **istradefylline** (1.0 mg/kg bw, green triangle, n = 4). Student T-test: $p < 0.05$*.

Tab. S2: Tissue biodistribution of radioactivity at different time point after i.v. injection of [^{18}F]**FLUDA** in CD-1 mice based on PET imaging.

	Uptake (SUV)*			
	**5 min**	**15 min**	**30 min**	**60 min**
**Blood**	1.59 ± 0.41	1.02 ± 0.16	0.61 ± 0.21	0.51 ± 0.21
**Heart**	0.57 ± 0.13	0.32 ± 0.05	0.20 ± 0.03	0.17 ± 0.04
**Small intestine**	6.19 ± 1.89	23.81 ± 8.91	40.11 ± 15.70	49.18 ± 24.48
**Spleen**	0.51 ± 0.18	0.33 ± 0.17	0.22 ± 0.13	0.20 ± 0.10
**Liver**	5.19 ± 1.50	2.89 ± 0.60	1.54 ± 0.71	1.20 ± 0.75
**Kidney**	1.20 ± 0.40	0.83 ± 0.64	0.77 ± 0.82	0.80 ± 0.87
**Muscle**	0.34 ± 0.09	0.21 ± 0.05	0.12 ± 0.02	0.10 ± 0.02
**Brain**	0.34 ± 0.13	0.22 ± 0.05	0.11 ± 0.02	0.05 ± 0.02

*Uptake values are represented as SUV mean ± S.D. (n = 4).

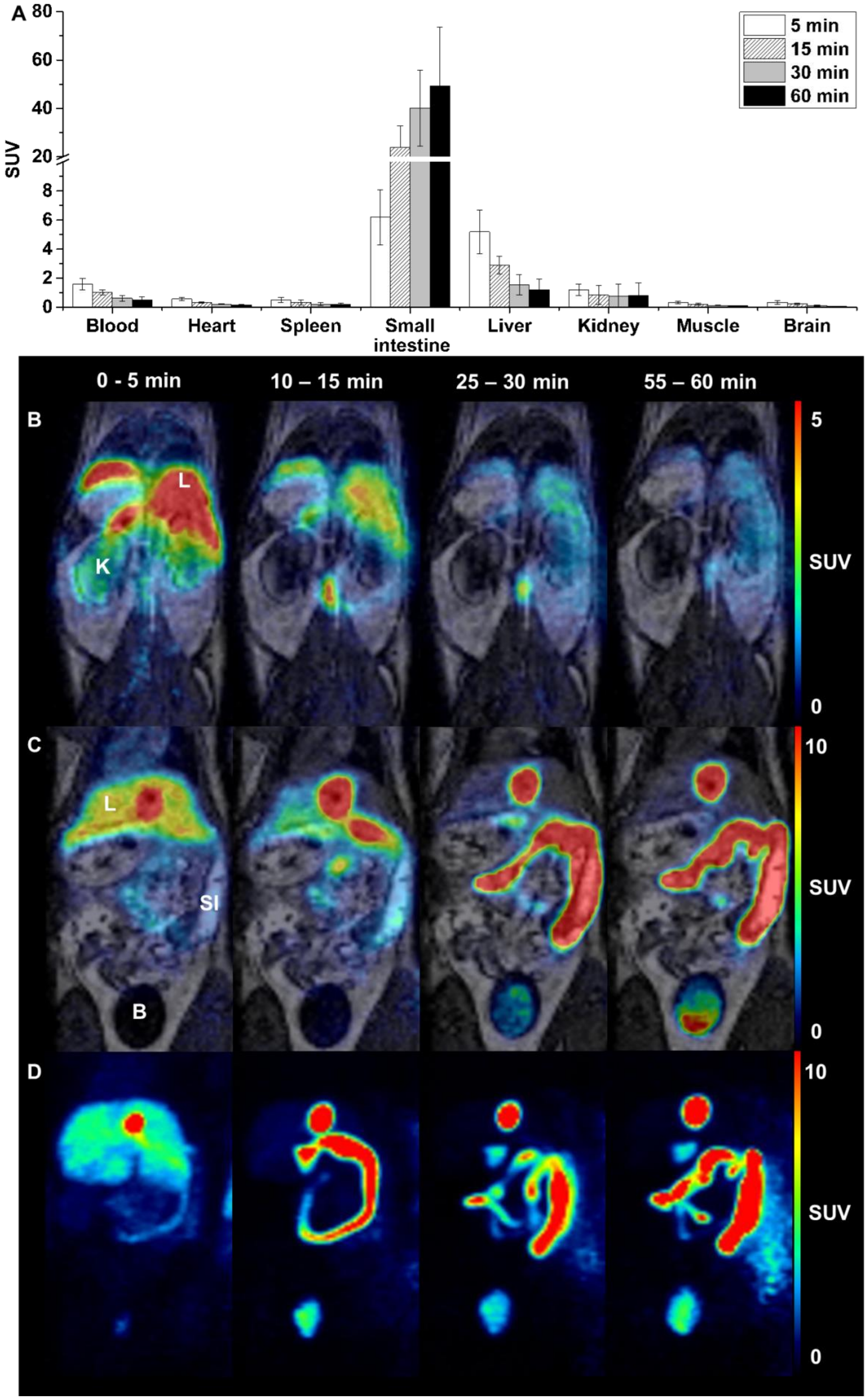

Fig. S9: (**A**) Biodistribution of [^{18}F]**FLUDA** in CD-1 mice at different time points based on PET imaging (n = 4); Representative horizontal merged PET/MRI images of [^{18}F]**FLUDA** biodistribution focused on (**B**) kidneys and liver and (**C**) small intestine and bladder, and (**D**) corresponding maximal intensity projection (MIP). K: kidneys; L: liver; SI: small intestine; B: bladder.

References

1. Barret O, Hannestad J, Alagille D, Vala C, Tavares A, Papin C, et al. Adenosine 2A receptor occupancy by tozadenant and preladenant in rhesus monkeys. *J Nucl Med.* **2014**;55:1712-8. doi:10.2967/jnumed.114.142067.

2. Brooks DJ, Doder M, Osman S, Luthra SK, Hirani E, Hume S, et al. Positron emission tomography analysis of [^{11}C]KW-6002 binding to human and rat adenosine A_{2A} receptors in the brain. *Synapse*. **2008**;62:671-81. doi:10.1002/syn.20539.

3. Hesk D, Borges S, Dumpit R, Hendershot S, Koharski D, McNamara P, et al. Synthesis of ^{3}H, $^{2}H_4$, and ^{14}C-MK 3814 (preladenant). *J Labelled Comp Radiopharm.* **2017**;60:194-9. doi:10.1002/jlcr.3490.

4. Perera S, Piwnica-Worms D, Alauddin MM. Synthesis of a ^{18}F-labeled ceritinib analogue for positron emission tomography of anaplastic lymphoma kinase, a receptor tyrosine kinase, in lung cancer. *J Labelled Compd Rad*. **2016**;59:103-8.

5. Baraldi PG, Borea PA. Adenosine A_3 receptor modulators. US patent application. 2002. US6358964B1.

6. Neustadt BR, Hao J, Liu H, Boyle CD, Chackalamannil S, Shah UG, et al. Pyrazolo-[4,3-*e*]-1,2,4-triazolo-[1,5-*c*]-pyrimidine adenosine A_{2a} receptor antagonists. PCT patent application. 2010. WO2005103055A1

7. Shinkre BA, Kumar TS, Gao ZG, Deflorian F, Jacobson KA, Trenkle WC. Synthesis and evaluation of 1,2,4-triazolo[1,5-*c*]pyrimidine derivatives as A_{2A} receptor-selective antagonists. *Bioorg Med Chem Lett.* **2010**;20:5690-4. doi:10.1016/j.bmcl.2010.08.021.

8. Schröder S, Lai TH, Toussaint M, Kranz M, Chovsepian A, Shang Q, et al. PET Imaging of the adenosine A_{2A} receptor in the rotenone-based mouse model of Parkinson's disease with [^{18}F]FESCH synthesized by a simplified two-step one-pot radiolabeling strategy. *Molecules.* **2020**;25:1633.

9. Wenzel B, Liu J, Dukic-Stefanovic S, Deuther-Conrad W, Teodoro R, Ludwig FA, et al. Targeting cyclic nucleotide phosphodiesterase 5 (PDE5) in brain: Toward the development of a PET radioligand labeled with fluorine-18. *Bioorg Chem.* **2019**;86:346-62. doi:10.1016/j.bioorg.2019.01.037.

10. Ludwig FA, Fischer S, Houska R, Hoepping A, Deuther-Conrad W, Schepmann D, et al. In vitro and in vivo Human Metabolism of (S)-[^{18}F]Fluspidine - A Radioligand for Imaging sigma1 Receptors With Positron Emission Tomography (PET). *Front Pharmacol.* **2019**;10:534. doi:10.3389/fphar.2019.00534.

11. Brust P, Patt JT, Deuther-Conrad W, Becker G, Patt M, Schildan A, et al. In vivo measurement of nicotinic acetylcholine receptors with [^{18}F]norchloro-fluoro-homoepibatidine. *Synapse.* **2008**;62:205-18.

12. Teodoro R, Scheunemann M, Deuther-Conrad W, Wenzel B, Fasoli FM, Gotti C, et al. A promising PET tracer for imaging of α_7 nicotinic acetylcholine receptors in the brain: Design, synthesis, and in vivo evaluation of a dibenzothiophene-based radioligand. *Molecules.* **2015**;20:18387-421. doi:10.3390/molecules201018387.

13. Ritawidya R, Wenzel B, Teodoro R, Toussaint M, Kranz M, Deuther-Conrad W, et al. Radiosynthesis and biological investigation of a novel fluorine-18 labeled benzoimidazotriazine-based radioligand for the imaging of phosphodiesterase 2A with positron emission tomography. *Molecules.* **2019**;24:4149.
14. Sattler B, Kranz M, Wenzel B, Jain NT, Moldovan R-P, Toussaint M, et al. Preclinical incorporation dosimetry of [^{18}F]FACH - A novel ^{18}F-labeled MCT1/MCT4 lactate transporter inhibitor for imaging cancer metabolism with PET. *Molecules.* **2020**;25:2024.
15. Flohr A, Moreau JL, Poli S, Riemer C, Steward L. 4-Hydroxy-4-methyl-piperidine-1-carboxylic acid (4-methoxy-7-morpholin-4-yl-benzothiazol-2-yl)-amide. PCT patent application. 2005. WO2005116026A1.
16. Shimada J, Koike N, Nonaka H, Shiozaki S, Yanagawa K, Kanda T, et al. Adenosine A_{2A} antagonists with potent anti-cataleptic activity. *Bioorg Med Chem Lett.* **1997**;7:2349-52.
17. Pretorius J, Malan SF, Castagnoli N, Jr., Bergh JJ, Petzer JP. Dual inhibition of monoamine oxidase B and antagonism of the adenosine A_{2A} receptor by (*E*,*E*)-8-(4-phenylbutadien-1-yl)caffeine analogues. *Bioorg Med Chem.* **2008**;16:8676-84. doi:10.1016/j.bmc.2008.07.088.
18. Cristalli G, Eleuteri A, Vittori S, Volpini R, Lohse MJ, Klotz KN. 2-Alkynyl derivatives of adenosine and adenosine-5'-N-ethyluronamide as selective agonists at A_2 adenosine receptors. *J Med Chem.* **1992**;35:2363-8. doi:10.1021/jm00091a003.
19. Kase H, Aoyama S, Ichimura M, Ikeda K, Ishii A, Kanda T, et al. Progress in pursuit of therapeutic A_{2A} antagonists: the adenosine A_{2A} receptor selective antagonist KW6002: research and development toward a novel nondopaminergic therapy for Parkinson's disease. *Neurology.* **2003**;61:S97-100. doi:10.1212/01.wnl.0000095219.22086.31.
20. Sihver W, Schulze A, Wutz W, Stusgen S, Olsson RA, Bier D, et al. Autoradiographic comparison of in vitro binding characteristics of various tritiated adenosine A_{2A} receptor ligands in rat, mouse and pig brain and first ex vivo results. *Eur J Pharmacol.* **2009**;616:107-14. doi:10.1016/j.ejphar.2009.06.025.
21. Poucher SM, Keddie JR, Singh P, Stoggall SM, Caulkett PW, Jones G, et al. The in vitro pharmacology of ZM241385, a potent, non-xanthine A_{2a} selective adenosine receptor antagonist. *Br J Pharmacol.* **1995**;115:1096-102. doi:10.1111/j.1476-5381.1995.tb15923.x.
22. de Zwart M, Vollinga RC, Beukers MW, Sleegers DF, von Frijtag Drabbe Künzel JK, de Groote M, et al. Potent antagonists for the human adenosine A_{2B} receptor. Derivatives of the triazolotriazine adenosine receptor antagonist ZM241385 with high affinity. *Drug Dev Res.* **1999**;48:95-103. doi:10.1002/(sici)1098-2299(199911)48:3<95::aid-ddr1>3.0.co;2-b.
23. Ongini E, Dionisotti S, Gessi S, Irenius E, Fredholm BB. Comparison of CGS 15943, ZM241385 and SCH58261 as antagonists at human adenosine receptors. *N-S Arch Pharmacol.* **1999**;359:7-10. doi:10.1007/pl00005326.
